# BranchMatch: point cloud registration for individual apple trees with limited overlap based on local structure characteristics

**DOI:** 10.3389/fpls.2025.1616611

**Published:** 2025-10-09

**Authors:** Ning Wang, Yuheng Cao, Kai Zhang, Shuting Xu, Ruifang Dong, Feng Kang, Man Zhang, Bojia Chi, Yanlong Miao, Yaxiong Wang

**Affiliations:** ^1^ Key Lab of State Forestry and Grassland Administration for Forestry Equipment and Automation, School of Technology, Beijing Forestry University, Beijing, China; ^2^ State Key Laboratory of Efficient Production of Forest Resources, School of Technology, Beijing Forestry University, Beijing, China; ^3^ Key Lab of Smart Agriculture System Integration, Ministry of Education, China Agricultural University, Beijing, China; ^4^ Beijing Miyun District Landscape and Greening Service Center, Beijing, China

**Keywords:** individual tree, key branch segments, low-overlap, terrestrial laser scanning, 3D reconstruction

## Abstract

Point cloud registration is a critical technology for 3D reconstruction and personalized management of fruit trees. While ensuring the accuracy and completeness of 3D point cloud reconstruction, the simplest and most efficient approach is to acquire and register point clouds from two stations separated by 180°. For this, we propose BranchMatch, a low-overlap viewpoints acquisition and registration method tailored for tall-spindle individual apple trees during dormancy. The method requires only two point clouds captured from stations 180° apart. Then, it leverages key branch segments in a single viewpoint, utilizing their spatial and geometric structure features in combination with a dynamically weighted feature discriminant function to perform feature matching and initial rigid-body transformation under low overlap conditions. Subsequently, an iterative closest point algorithm, enhanced with local feature matching optimization based on the tree-specific point cloud, is applied to refine the registration and prevent over-registration. Experiments conducted on multiple individual apple trees with two low-overlap point clouds (180° apart) demonstrate a registration success rate of 90%. Compared to the spherical markers registration method, BranchMatch achieves average rotation and translation errors of 1.93 mrad and 4.33 mm, respectively, with a pointwise error of 2.70 mm. Furthermore, compared to multi-site high-overlap registration methods under similar conditions, BranchMatch significantly reduces computational costs while maintaining registration accuracy and reconstruction completeness, highlighting its efficiency and reliability in individual tree registration.

## 1 Introduction

The apple (*Malus domestica*) is one of the most economically significant and widely cultivated fruit crops globally, making advancements in its orchard management critical to the agricultural economy (Vasylieva and Harvey, 2021; Wang and Liu, 2022). In modern apple production, the tall-spindle architecture has become the dominant tree form, as it enables the high-density planting strategies essential for maximizing fruit quality and yield (Robinson et al., 2006; Karkee et al., 2014; Ho et al., 2024). Effective management of these trees relies heavily on a detailed understanding of their underlying branching structure, which informs key operations such as strategic pruning and yield forecasting. The dormancy period offers a unique opportunity for this kind of structural analysis, as the absence of leaves and fruit reveals the complete woody framework of the tree—making it the optimal time for both assessment and pruning interventions (Chattopadhyay et al., 2016; He and Schupp, 2018; Kolmanič et al., 2021).

To address the need for accurate and efficient structural analysis of apple trees, 3D reconstruction based on Light Detection and Ranging (LiDAR) technology has emerged as a powerful tool (Dassot et al., 2011; Miller et al., 2015; Zhang et al., 2023). While mobile laser scanning (MLS) systems—such as UAV-based or backpack-mounted devices—are effective for rapid, large-scale orchard mapping, the millimeter-level accuracy required for detailed individual-tree analysis remains challenging to achieve with these systems (Liang et al., 2016; Calders et al., 2020; Wang L. et al., 2023). In contrast, Terrestrial Laser Scanning (TLS) provides superior data fidelity and resolution, making it the most widely accepted and reliable choice for fine-scale, branch-level reconstruction tasks (Shimizu et al., 2022; Zhang et al., 2024).

To ensure the accuracy and completeness of 3D reconstruction, it is common practice to register point clouds acquired from different viewpoints (Monji-Azad et al., 2023). However, the complex orchard environment introduces challenges such as occlusions and structural deformations, making accurate and efficient point cloud registration for fruit trees a persistent challenge.

The most reliable registration method involves marker-based approaches, where spherical markers are manually placed before data acquisition, requiring all viewpoints to capture these markers in their point clouds. Key points are extracted via fitting calculations, and correspondences (e.g., distances, angles) between markers across viewpoints are used for registration (Wilkes et al., 2017; Zhou et al., 2020). While accurate, this method increases operational complexity due to manual marker placement.

To mitigate this limitation, marker-free techniques utilize sensors or specially designed platforms to record scan station positions and orientations (Henning and Radtke, 2008; Yang R. et al., 2020). These approaches improve efficiency and, can be applied to multitemporal assessments and permanent monitoring of trees within the same plots. Nevertheless, both marker-based and position-based methods increase acquisition burdens.

In scenarios lacking standardized targets or positioning tools, registration must rely on the point cloud data of the scene itself (Cheng et al., 2018), necessitating robust algorithmic frameworks. One approach involves using point cloud feature descriptors to match key points based on the inherent characteristics of the data. Zhang et al. (2021) employed the Fast Point Feature Histogram (FPFH) to capture the geometric distinctiveness of individual trees, enabling coarse registration of multi-view point clouds by matching similar feature descriptors across viewpoints. Peng et al. (2023) further improved FPFH-based registration for airborne and terrestrial point clouds, combining feature correlation scoring with Bhattacharyya distance and employing Random Sample Consensus (RANSAC) to select reliable matching pairs. While these methods are robust and flexible, their performance heavily relies on feature quality, making them sensitive to noise and computationally demanding. Another approach leverages the geometric features of trees combined with spatial information to establish correspondences. Bucksch and Khoshelham (2013) extracted global skeletons from fruit tree point clouds, using them to map points in the source point cloud to line segments in the target point cloud, followed by transformation estimation through distance minimization. Zhou et al. (2014) further defined a distance measure and mapping cost function between two skeleton segments, refining them using the Gauss-Newton method to complete the registration. These methods provide a domain-specific strategy for tree point cloud registration, enabling semi-automatic or automatic alignment through skeleton points or lines. That said, they require complete skeleton extraction, which is challenging due to occlusion, density variations, and complex branching structures.

Both approaches eliminate the need for external markers or prior information but require high-quality single-view data and typically depend on multi-view acquisitions with substantial overlap (≥3 viewpoints). These approaches utilize geometric or textural features from overlapping regions to establish correspondences and merge partial scans into complete models (Yang et al., 2022). However, in orchard environments, physical obstructions (e.g., irrigation systems, support structures), limited scanner mobility, and operational efficiency demands often hinder multi-view high-overlap data collection (Shao et al., 2020), necessitating alternative low-overlap registration strategies.

What’s more, in recent years, deep learning-based methods have emerged as another powerful paradigm for point cloud registration. These approaches leverage neural networks to learn salient features and correspondences directly from data, often achieving impressive performance on general-purpose benchmarks (Dai et al., 2022; Cheng et al., 2024). However, their practical application to the high-fidelity registration of individual trees in orchard environments currently faces several significant limitations. First, these models are highly data-dependent, requiring large-scale, annotated datasets for training that are not readily available for high-resolution, multi-view tree scans (Cheng et al., 2018; Lv et al., 2024). Furthermore, their ability to generalize is a key concern; models trained on generic 3D objects may not perform well on the unique and complex geometric structures of dormant trees. This challenge is particularly acute in the extreme low-overlap scenarios addressed by our study (Yang H. et al., 2020; Wang et al., 2024). Finally, the high computational costs associated with training, coupled with a lack of sufficient validation in multi-station orchard scenarios (Dong et al., 2020; Deng et al., 2021), make classical geometry-based methods a more practical and reliable choice for our specific application at present.

In summary, in terms of field efficiency, the optimal strategy is to capture data from only two diametrically opposed (180°) stations. This approach, however, presents the most extreme registration challenge due to minimal data overlap. The difficulty of reliably automating this process has largely prevented the adoption of this otherwise ideal workflow. Overcoming this registration bottleneck is therefore a key step toward making high-precision 3D reconstruction more practical. The inherent limitations of this low-overlap scenario introduce several critical challenges:

1. A more robust feature extraction and matching method is needed for the geometrically ambiguous branch structures encountered with sparse overlap between diametrically opposed viewpoints.
2. A novel registration approach is required to minimize reliance on the overlap between viewpoints of tree-shaped point clouds.
3. A lack of individual tree point cloud datasets hinders registration accuracy evaluation under varying overlap conditions.

To address these challenges, this study focuses on acquiring high-precision 3D point cloud data of tall-spindle individual apple trees using terrestrial laser scanning (TLS). By leveraging the structural features of key parts of the tree, we aim to achieve marker-free, low-overlap registration for individual apple trees, enabling high-accuracy 3D reconstruction and advancing fruit tree management. The main contributions are as follows:

1. A feature matching method based on local spatial-geometric branch features, incorporating a dynamically weighted discriminant function to reliably identify corresponding branch segments under low-overlap conditions.
2. An automatic registration method using structural axes and central points of key tree components, enabling accurate reconstruction from two 180° viewpoints.
3. The Apple-Trees benchmark dataset, comprising 10 trees captured from 40 viewpoints, covering diverse tall-spindle morphologies for registration evaluation.

## 2 Benchmark dataset

### 2.1 Data acquisition

**Figure 1:**
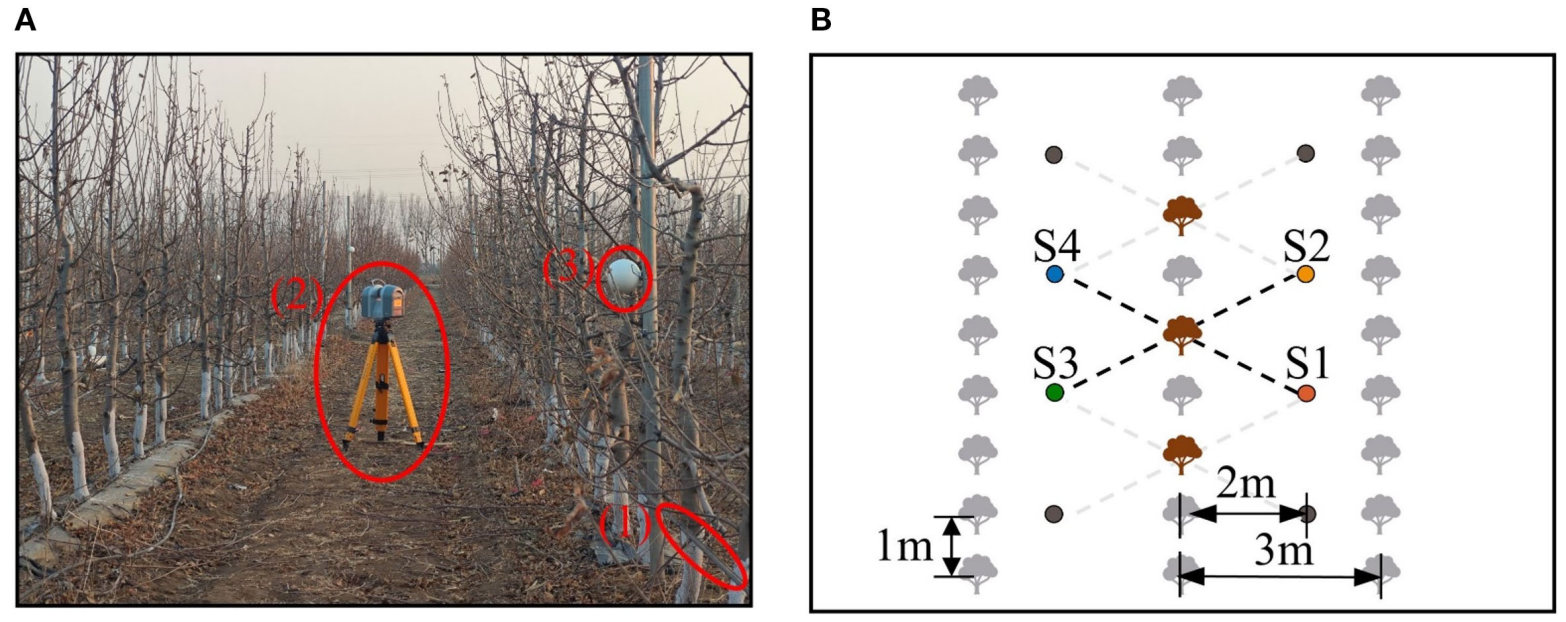
Data acquisition environment and setup. **(A)** Orchard environment and equipment. **(B)** Schematic of station positions. In **(A)**, (1), (2), and (3) represent the drip irrigation tape, TX8 terrestrial LiDAR, and spherical markers, respectively. In **(B)**, the brown objects represent target trees, and the solid dots denote stations. Data collection was performed using interval selection and station reuse, ensuring that each tree was captured from four viewpoints, with opposite viewpoints separated by 180°.

The Apple-Trees dataset was collected from a commercial orchard in Shunyi District, Beijing, China (40.21°N, 116.54°E). The orchard features Aztec Fuji apple trees, a tall-spindle variety commonly grown in northern orchards. The 5–7 years-old trees, averaging 300–350 cm in height, were planted in rows with 1 m spacing between trees and 3 m between rows. Drip irrigation tapes were installed at 0.5 m above the ground to facilitate precise irrigation, as shown in **Figure 1**.

Data acquisition was conducted in March 2024 during the dormancy period to avoid interference from fruits and dense foliage (**Figure 1A**). The Trimble TX8 terrestrial LiDAR was selected as the data acquisition device for the experiment, as similar terrestrial LiDAR systems are widely recognized as effective and precise tools for acquiring point cloud data during high-precision 3D reconstruction (Guan et al., 2020; Sun et al., 2021). The device has a 360° × 317°field of view, a measurement range of 0.6–120 m, and an accuracy of up to 2 mm. Double leveling was achieved using both external and built-in electronic leveling bubbles.

To compile the dataset, 10 apple trees were randomly selected from three rows using interval selection (**Figure 1B**). For each selected tree, data were collected from four stations positioned in a rectangular arrangement around the tree. Stations were positioned approximately 2 m from the tree rows and 2.3 m from the selected trees, considering the orchard layout. Station pairs S1-S4 and S2-S3 provided low-overlap 180° viewpoints, while S1-S2 and S1-S3 offered high-overlap viewpoints for algorithm evaluation.

The raw data collected by the instrument was processed using Trimble RealWorks software, where point clouds were extracted via spatial sampling at a 2 mm resolution. Since the collected raw data included multiple trees, extensive ground areas, and other environmental features, it was necessary to manually extract the regional point cloud of each target tree to enable a fair and independent evaluation of the algorithm’s performance on the tree structure itself. To minimize the impact on the practical application process, this manual step was limited to a simple regional segmentation to obtain all points within the single fruit tree’s area (including the ground points). Additionally, to ensure the evaluation focused solely on the tree’s complex geometry and to enhance the method’s generalizability, the geometrically simple and distinct drip tape point clouds were also manually removed.

**Figure 2:**
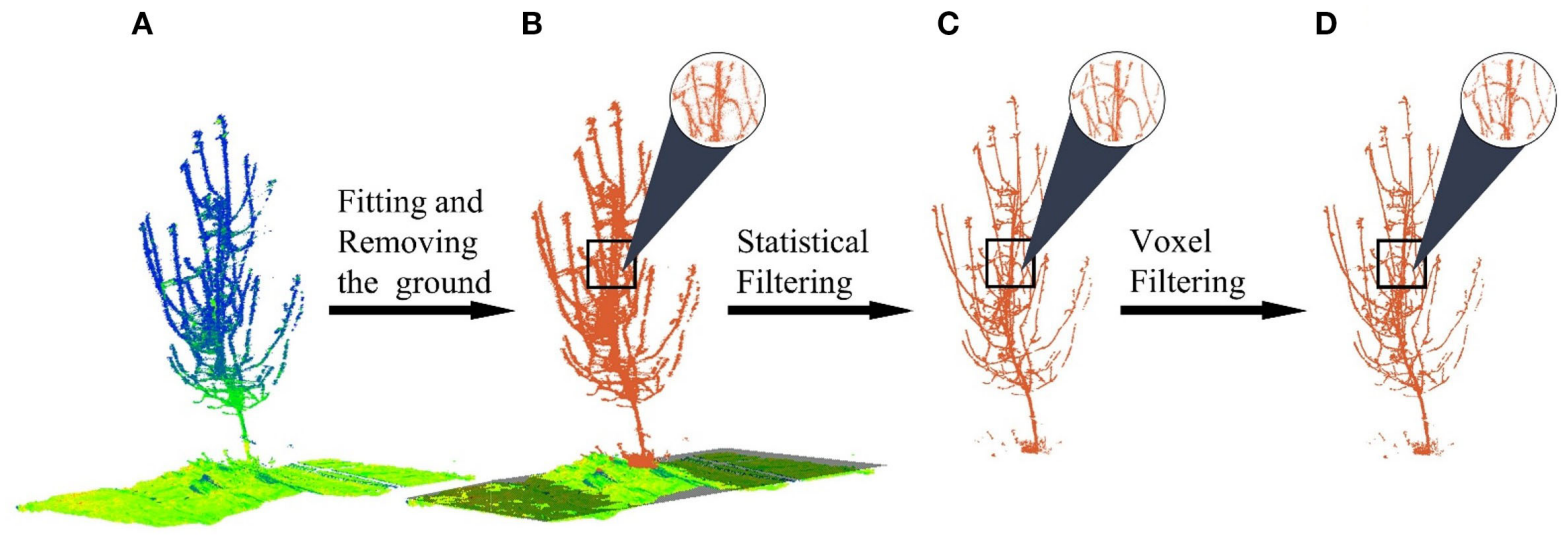
Point cloud preprocessing. **(A)** Raw point. **(B)** Ground plane fitted and removed. **(C)** Statistical filtering. **(D)** Voxel filtering.

Following obtaining the point cloud of the experimental region (**Figure 2A**), a series of automated preprocessing steps are sequentially applied to prepare the data for the core matching algorithm, as illustrated in **Figure 2**. The first and most critical step is to establish a consistent spatial reference by fitting a ground plane using the RANSAC method (Fischler and Bolles, 1981). This plane serves as the basis for all subsequent height-dependent feature calculations.

Once this coordinate system is established, the ground points are removed to reduce computational load and focus the analysis on the tree’s structure (**Figure 2B**). Subsequently, a statistical outlier removal filter is applied to eliminate sparse noise points that may have resulted from sensor errors or environmental factors. The filter was configured with a number of neighbors k = 5 and a standard deviation multiplier of 0.1 (**Figure 2C**). Finally, to enhance computational efficiency for the subsequent registration steps, the point cloud is downsampled using a voxel grid filter with a voxel size of 8 mm, which standardizes the point density while preserving the essential geometric details of the trunk and branches (**Figure 2D**).

### 2.2 Ground truth

To establish baseline alignment data, spherical markers were additionally deployed as a ground truth method for point cloud registration (Liang et al., 2018). Specifically, markers were arranged to cover the region of interest (**Figure 1A**), and precisely leveled LiDAR scans captured all scene elements, including the markers. The collected point clouds were imported into Trimble RealWorks, where spherical markers were automatically identified, and their centers were fitted to establish correspondences. The software calculated an optimal transformation matrix (rotation and translation) by minimizing alignment errors, producing a ground truth dataset with an average distance error of 0.59 mm. This accuracy serves as the benchmark for subsequent experimental evaluations.

What’s more, the complete point clouds, registered from all four stations using this ground truth method, also serve as the baseline reference for deriving the structural parameters of each sample tree. **Table 1** provides detailed information for each tree in the Apple-Trees dataset, including statistics on the point clouds and these key structural parameters.

**Table 1:** Statistics on the Apple-Trees benchmark dataset.

Tree ID	Tree height (cm)	Basal trunk diameter (cm)	No. of primary branches	Max. branch diameter (cm)	Avg. branch diameter (cm)	Number of points of each scan (excluding ground points)			
						S1	S2	S3	S4
#1	351	6.3	14	3.1	2.1	255,180	264,954	265,352	278,387
#2	353	7.9	11	3.3	2.0	311,608	335,566	310,395	290,280
#3	314	6.4	18	2.5	1.7	211,552	231,864	253,044	246,309
#4	336	5.1	11	2.7	1.8	253,859	289,802	258,970	232,020
#5	327	6.2	9	1.7	1.4	185,871	164,238	188,364	161,512
#6	368	6.3	9	2.5	1.8	200,625	233,390	208,178	162,251
#7	366	5.6	11	2.6	1.8	240,547	242,450	289,745	293,047
#8	247	5.7	8	2.8	1.7	104,469	109,095	136,006	115,101
#9	287	7.3	10	2.2	1.6	192,990	207,089	204,775	214,273
#10	334	6.3	10	3.0	1.8	273,238	273,121	268,062	306,510

Basal Trunk Diameter was determined from a horizontal cross-section at a height of 0.25 m above the fitted ground plane; Primary branches were defined and counted as first-order branches with a basal diameter > 1cm; Avg. Branch Diameter is the average of these measured basal diameters.

## 3 Methodology

**Figure 3:**
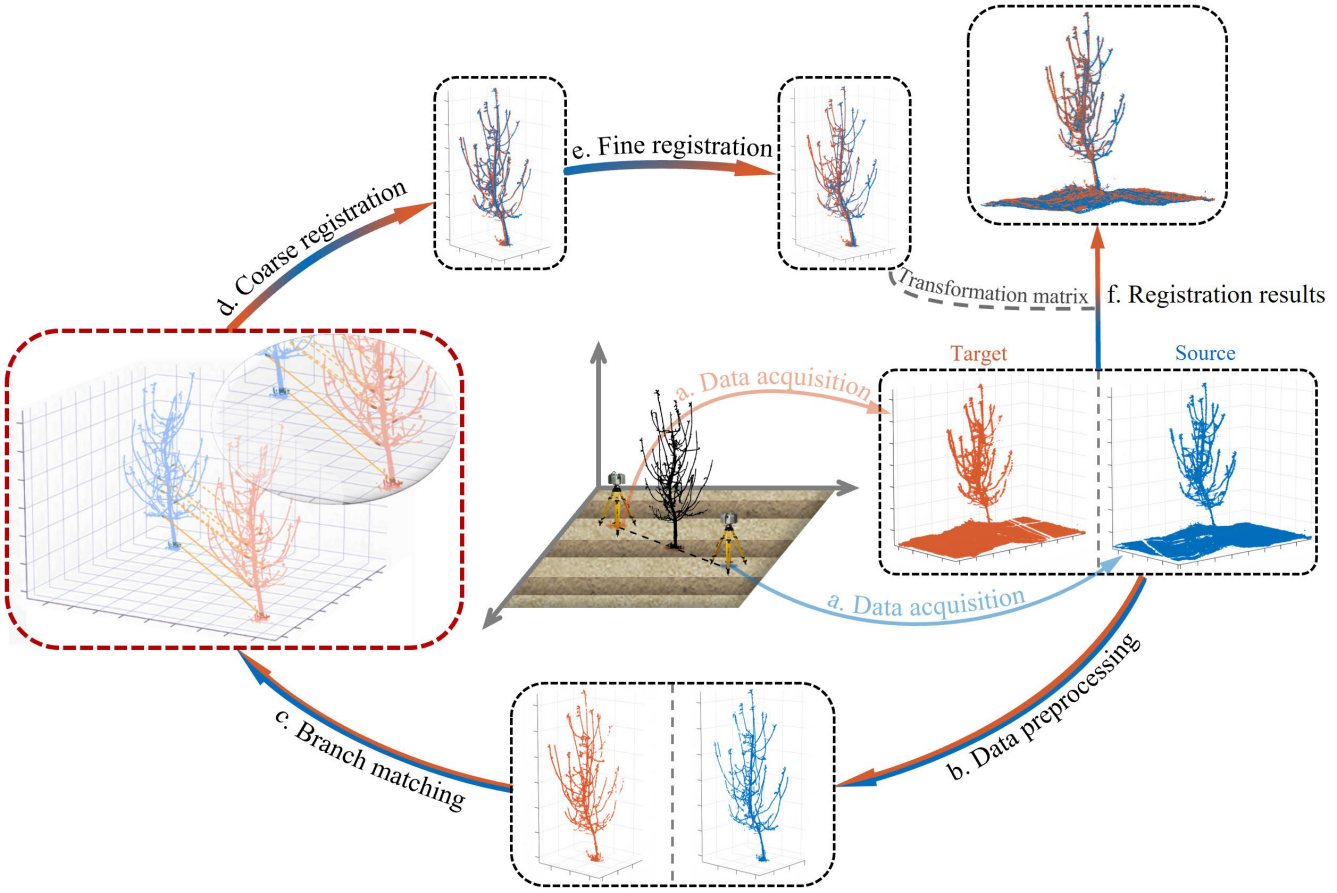
Workflow of the BranchMatch method. The process includes **(a)** Data acquisition, **(b)** Data preprocessing, **(c)** Branch matching, **(d)** Coarse registration, **(e)** Fine registration and **(f)** Registration results. The transformation matrix is computed through steps a.-e., and the final results aligns the source and target point clouds **(f)**.

The BranchMatch method requires two 180°-separated point clouds of an individual tree as input and outputs a rigid-body transformation matrix for registration, enabling 3D reconstruction. The process consists of three main steps: branch matching, coarse registration, and fine registration. The overall procedure is illustrated in **Figure 3**.

### 3.1 Branch matching

Point cloud registration typically relies on identifying correspondences within overlapping regions (Liu et al., 2017). However, in diametrically opposed 180° scans, these regions are confined to sparse transitional boundaries—narrow zones where front and rear projections partially intersect. These sparse common points in such boundaries exhibit feature ambiguity due to occlusion and geometric complexity, limiting the effectiveness of both conventional methods (e.g., FPFH) and learning-based approaches (Fu et al., 2023).

To address this, the proposed method eliminates dependency on explicit overlap by leveraging the structural consistency of trees. By focusing on these anatomically consistent components, such as trunks and primary branches, which are typically observable from both views despite low overlap, the algorithm locates corresponding structural parts in each point cloud and constructs robust initial alignments under challenging low-overlap conditions.

For the trunk, the same parts across viewpoints can be identified using height above ground. However, relying solely on the trunk often fails to meet 3D registration requirements. Therefore, branch morphological and spatial distribution characteristics are utilized to assist registration. Branches exhibit significant morphological differences and distinctive spatial distributions, providing a critical basis for matching point clouds from different perspectives.

Describing trunks and branches typically requires referencing the ground as a baseline and employing 2D circular or 3D cylindrical fitting to extract structural information (Pfeifer et al., 2004; Xu et al., 2022). However, trunks and branches often appear as ellipses or irregular shapes in thin cross-sections, and noise complicates precise differentiation. While 2D circle fitting is computationally intensive and error-prone, cylindrical fitting, which incorporates multi-layer information, improves stability and accuracy, outperforming single-layer fitting (Raumonen et al., 2013; Li et al., 2022). Thus, this paper adopts cylindrical fitting for better localization.

Here we detail the branch matching process, covering the segmentation of key branch segments, the filtering of potential pairing branches, and the final selection of the best match.

**Figure 4:**
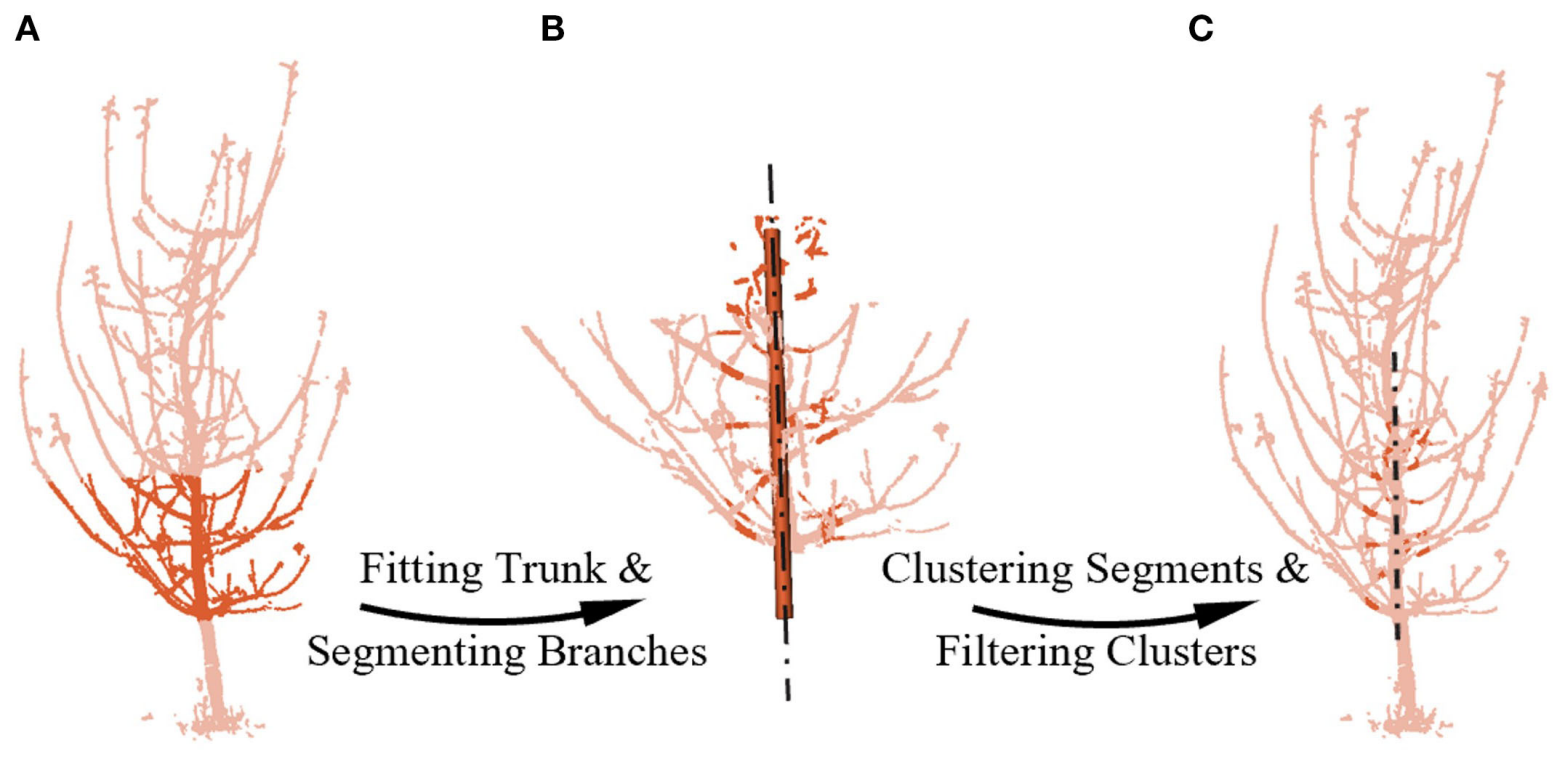
Segmenting and filtering potential pairing branches. **(A)** Mid-to-lower point cloud extraction. **(B)** Fitting the mid-to-lower trunk and segmenting branchs by distance from the cylinder axis. **(C)** Filtering potential pairs using cluster size and inlier ratio. The chain lines represents the axis line obtained by fitting.

Firstly, branch segments in the same key region of both point clouds are segmented. This initial step is designed to simplify the complex matching problem by focusing on short, geometrically simple segments rather than the entire intricate branching structure. Specifically, we isolate the tree’s middle-lower sections based on height above the ground (**Figure 4A**), where the trunk and branches are thicker and less affected by wind, and the trunk reflects the tree’s overall growth trend. Given the trunk’s larger radius, we can use RANSAC to fit a cylinder (Fischler and Bolles, 1981) and identify the trunk, obtaining its axis. This axis then serves as a reference to segment the branch segments within a specific distance (**Figure 4B**).

To define the area of branch segments suitable for robust matching, we conducted field measurements and statistical analysis on the 5–7-year-old tall-spindle apple trees used in our study. Our analysis indicated that while branch-trunk junction shapes vary significantly in the region up to 0.07 m from the trunk, the zone between 0.07 m and 0.14 m is optimal. Within this region, branches consistently display more pronounced cylindrical characteristics with fewer secondary branches, which reduces geometric ambiguity for our matching algorithm. In addition, as these segments are located closer to the trunk, they are less susceptible to wind-induced motion, further improving their geometric stability. This region is also more likely to contain branches segments that are both visible from both opposing viewpoints, thereby reducing mismatches due to single-view visibility in the 180° setup. Meanwhile, the standardized tall-spindle horticultural system, through continuous pruning, ensures that this key structural geometry remains highly consistent across the representative age range of our subjects and is less influenced by species-specific variations. Consequently, this region is identified as the key branch area for registration.

Secondly, potential pairing branches are filtered among the key branch segments obtained earlier. For the initial screening, the Euclidean clustering method (Miao et al., 2023) was employed to filter clusters based on point count. Larger clusters, which likely contain curved branch segments or secondary branch junctions, and smaller clusters, typically representing thinner branches or incomplete point clouds, were both excluded due to their poor conformity to cylindrical shapes. Next, cylindrical fitting was performed on clusters of moderate size, and the quality of the fit was evaluated by the proportion of inliers (Salehi et al., 2022; Martínez-Otzeta et al., 2023). Clusters with an inlier ratio below 30% were discarded, while those exceeding the threshold were labeled as “potential pairing branches.” Thus, we filtered potential pairing branches $S=\{s_{1},s_{2},\ldots ,s_{n}\}$ from the source point cloud and $T=\{t_{1},t_{2},\ldots ,t_{m}\}$ from the target point cloud, as illustrated in **Figure 4C**. At this stage, there are m×n possible pairing combinations between S and T .

**Figure 5:**
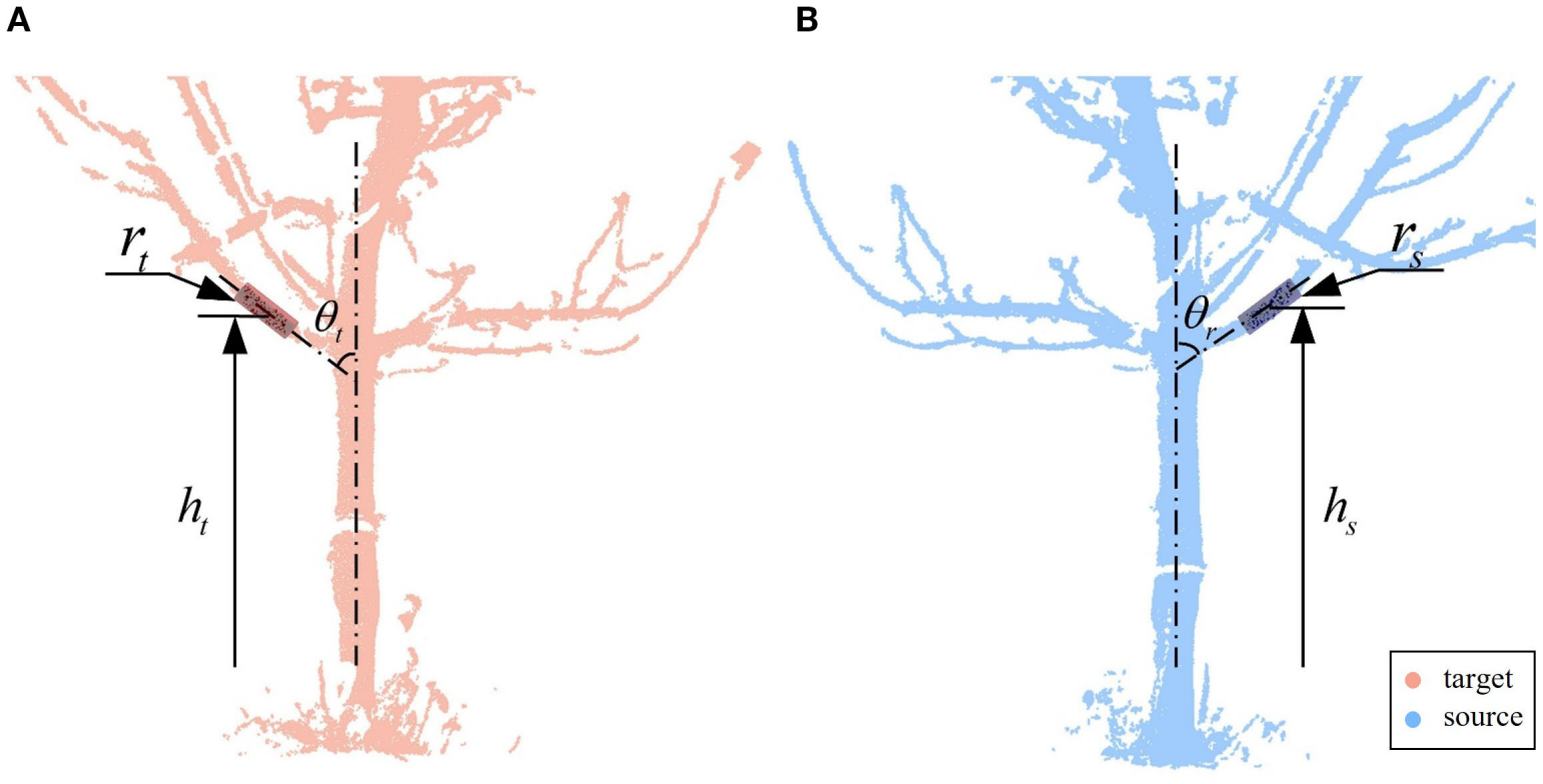
The best match. **(A)** Target point cloud branch parameters. **(B)** Source point cloud branch parameters. Branch segments are characterized using three feature parameters: r , θ , and h .

Finally, the best match is selected from the possible pairing combinations. To identify structural correspondences under low-overlap conditions, we introduce a branch matching strategy based on local geometric attributes. Specifically, three key features are extracted and quantified for each candidate branch segment: the radius r of the fitted branch cylinder, the angle θ between its axis and the trunk cylinder axis, and the height h of its centroid above the ground, as shown in **Figure 5**. These features jointly characterize the spatial and morphological properties of each branch, offering sufficient distinctiveness and consistency across viewpoints.

Due to inherent fitting errors arising from occlusions, noise, and viewpoint differences, residual discrepancies frequently exist between the feature values of truly corresponding branches. To account for this, we first compute the differences in radius, angle, and height for all m×n possible pairing combinations, and compare them against empirically determined thresholds ( $tol_{r}$ = 5 mm, $tol_{\theta }$ = 15°, $tol_{h}$ = 50 mm). These thresholds were established through data-informed tuning on a representative subset of manually annotated branch pairs. They are conservatively chosen to accommodate noise and fitting uncertainty while effectively eliminating structurally implausible matches. Only pairs satisfying all three threshold conditions are retained for further evaluation.

Although threshold-based filtering eliminates most implausible matches, finer ambiguities may still arise when multiple source branches fall within the threshold bounds of the same target branch. To resolve such cases, enforce one-to-one correspondence, and account for the differing sensitivities of the three features, we introduce a feature discriminant function with dynamic weights to compute a fused residual (Equation 1):


(1) $$score=(weight_{r}\frac{\Delta r}{tol_{r}})+(weight_{\theta }\frac{\Delta \theta }{tol_{\theta }})+(weight_{h}\frac{\Delta h}{tol_{h}})$$

where Δr , Δθ and Δh represent the residuals of the radius, angle, and height, respectively, such as $\Delta r=|r_{s}-r_{t}|$ for radius residual. The corresponding weighting factors are $weight_{r}$ , $weight_{\theta }$ and $weight_{h}$ , calculated as follows Equation 2:


(2) $$\left\{ \begin{array}{c} weight_{r}=-\frac{1}{weight_{sum}}\ln(\frac{\Delta r}{tol_{r}})\\ weight_{\theta }=-\frac{1}{weight_{sum}}\ln(\frac{\Delta \theta }{tol_{\theta }})\\ weight_{h}=-\frac{1}{weight_{sum}}\ln(\frac{\Delta h}{tol_{h}})\\ weight_{sum}=-\ln(\frac{\Delta r}{tol_{r}})-\ln(\frac{\Delta \theta }{tol_{\theta }})-\ln(\frac{\Delta h}{tol_{h}}) \end{array}\right.$$

The core idea of this dynamic weighting strategy is to assign higher weights to features with smaller residual, thereby emphasizing the contribution of geometrically consistent features to similarity assessment. Unlike static weighting, this adaptive strategy adjusts feature importance based on different structural configurations, enabling more effective differentiation between correct and false matches. Specifically, a normalized dynamic weighting strategy is employed using a negative logarithmic function. Each feature’s residual is normalized by dividing by its predefined threshold, and the negative logarithm of the normalized residual is applied to compute dynamic weights. The final score, which indicates fused residual of multiple features, is calculated using these weights, where lower scores correspond to smaller residual and higher similarity. Ultimately, for each branch in the target point cloud, only the source point cloud pairing with the smallest score is retained, ensuring a high-confidence one-to-one match.

As a final validation step, the quality of the cylindrical fit is used as a global confidence measure. Among all candidate pairs retained after scoring, the pair with the highest number of inlier points in its fitted cylinder is selected. This tie-breaking criterion ensures that the final correspondence used for transformation estimation is based on structurally robust and reliably measurable segments, adding an additional layer of stability to the matching process.

### 3.2 Coarse registration

The essence of point cloud registration is solving the transformation matrix T that aligns the source point cloud $\text{𝒫}=\left\{p_{i}^{*},1\leq i\leq N_{P}^{*}\right\}$ with the target point cloud $\text{𝒬}=\left\{q_{i}^{*},1\leq i\leq N_{q}^{*}\right\}$ . This process can be expressed by Equation 3:


(3) $$\left[\begin{array}{c} q_{i}^{*}\\ 1 \end{array}]=T[\begin{array}{c} p_{i}^{*}\\ 1 \end{array}\right]$$

where $p_{i}^{*}$ and $q_{i}^{*}$ represent the coordinates of the *i-*th point in the source and target point clouds, respectively. T , as a rigid-body transformation in space, is typically considered 6 degrees of freedom (DoF) transformation, consisting of 3-DoF for rotation and 3-DoF for translation.


(4) $$T=[\begin{array}{cc} R & t\\ 0 & 1 \end{array}]=[\begin{array}{cccc} R_{11} & R_{12} & R_{13} & t_{x}\\ R_{21} & R_{22} & R_{23} & t_{y}\\ R_{31} & R_{32} & R_{33} & t_{z}\\ 0 & 0 & 0 & 1 \end{array}]$$

Since most TLS devices, including the TLS used in this study, provide high accuracy in the horizontal direction (Cai et al., 2019; Zang et al., 2021), rotations around the X and Y axes can be neglected during the matrix solution, and the matrix T simplifies to $\dot{T}$ (Equation 5):


(5) $$\dot{T}=[\begin{array}{cc} R(\phi ) & t\\ 0 & 1 \end{array}]=[\begin{array}{cccc} \cos\phi & -\sin\phi & 0 & t_{x}\\ \sin\phi & \cos\phi & 0 & t_{y}\\ 0 & 0 & 1 & t_{z}\\ 0 & 0 & 0 & 1 \end{array}]$$

The simplified matrix $\dot{T}$ is a 4-DoF problem, and Wang X. et al. (2023) have also compared 6-DoF and 4-DoF estimation methods for TLS point cloud registration, with the latter demonstrating significantly higher accuracy. The transformation matrix $\dot{T}$ can be further decomposed into a 1-DoF rotation around the vertical axis and a 3-DoF translation in space. Specifically, rotational alignment in the XOY plane is achieved using the best matching branch, followed by translational alignment in 3D space using trunk information.

**Figure 6:**
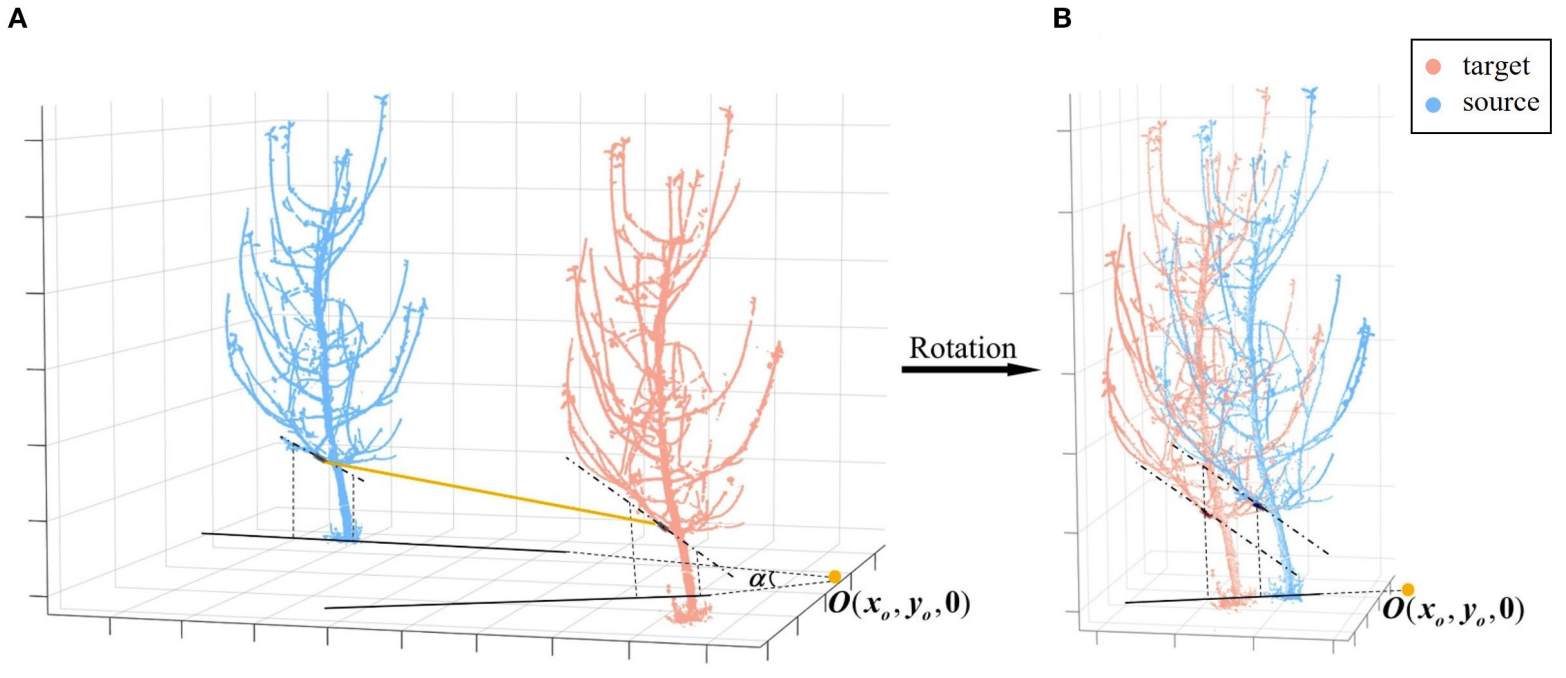
Coarse registration rotation. **(A)** Projection of the axes of matched branch segments. **(B)** Rotation to align the axes.


The first step is rotation in the XOY plane, i.e., around the vertical axis. In low-overlap viewpoints, the structural information of matched branches and trunks exhibits higher consistency. To leverage this, cylinders are fitted to the best matching branch, and their axes are extracted. These axes are then projected onto the XOY plane, with their intersection point denoted as $O(x_{o},y_{o},0)$ . The angle between the projected axes determines the required rotation angle α . Specifically, the center of rotation is first shifted from the origin to O . The source point cloud is then rotated around O , after which the rotated point cloud is translated back to the origin. Through this series of combined transformations, the transformation matrix can be calculated (Equation 6). As shown in **Figure 6**, this step aligns the two point clouds by rotation.

(6) $$T_{1}=[\begin{array}{cccc} \cos\alpha & -\sin\alpha & 0 & x_{o}(1-\cos(\alpha ))+y_{o}\sin(\alpha )\\ \sin\alpha & \cos\alpha & 0 & y_{o}(1-\cos(\alpha ))-x_{o}\sin(\alpha )\\ 0 & 0 & 1 & 0\\ 0 & 0 & 0 & 1 \end{array}]$$

**Figure 7:**
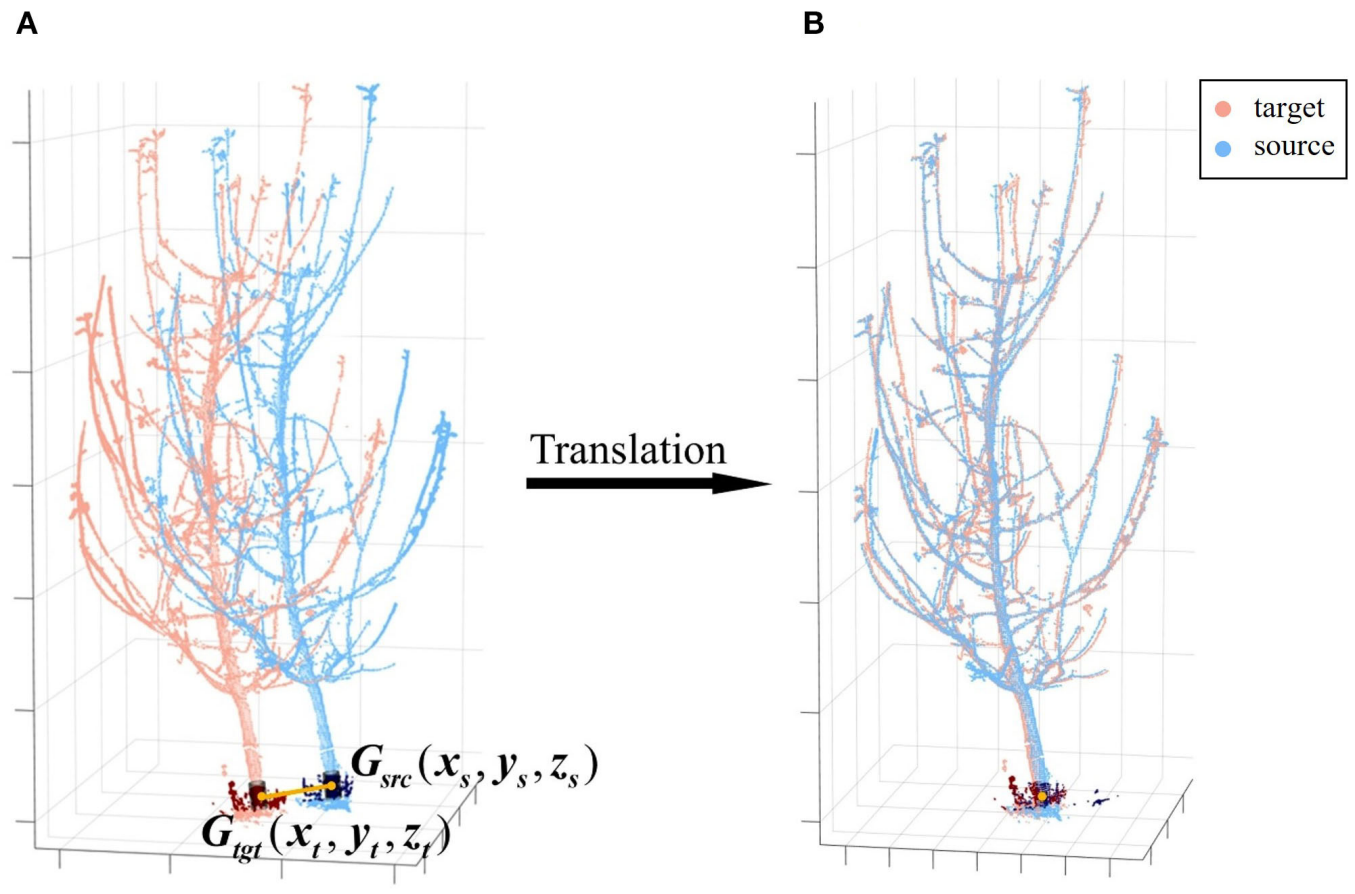
Coarse registration translation. **(A)** Center points of matched trunk segments. **(B)** Translation to align the center points. *G_src_* and *G_tgt​_* denote the geometric centers of the matched basal trunk segments for the source and target point clouds, respectively.


The next step is a straightforward translation operation, aligning corresponding points after rotational alignment. Given that the basal trunk generally lacks lateral branches, has a larger diameter, retains more complete point clouds, and approximates a cylinder, its structural center is selected as the reference for translation. Specifically, a trunk section at the same height is extracted from both viewpoints—at a height between 0.20 m and 0.30 m above the ground plane, a range chosen to avoid irregularities near the root collar while remaining below the first major branches—approximating different observations of the same bottom trunk. And then a cylindrical model is fitted to this section by RANSAC, and from its resulting axis, the geometric center of this trunk segment is computed, denoted as $G_{src}(x_{s},y_{s},z_{s})$ in the source point cloud and $G_{tgt}(x_{t},y_{t},z_{t})$ in the target point cloud (**Figure 7**). These centers serve as the corresponding points for translation. At this stage, the coordinate difference between the corresponding points represents the translation component of the transformation matrix, which can then be obtained (Equation 7):

(7) $$T_{2}=[\begin{array}{cccc} 1 & 0 & 0 & x_{t}-x_{s}\\ 0 & 1 & 0 & y_{t}-y_{s}\\ 0 & 0 & 1 & z_{t}-z_{s}\\ 0 & 0 & 0 & 1 \end{array}]$$

With this, the complete rotation translation matrix for coarse registration can be constructed as $T_{c}=T_{2}T_{1}$ .

### 3.3 Fine registration

**Figure 8:**
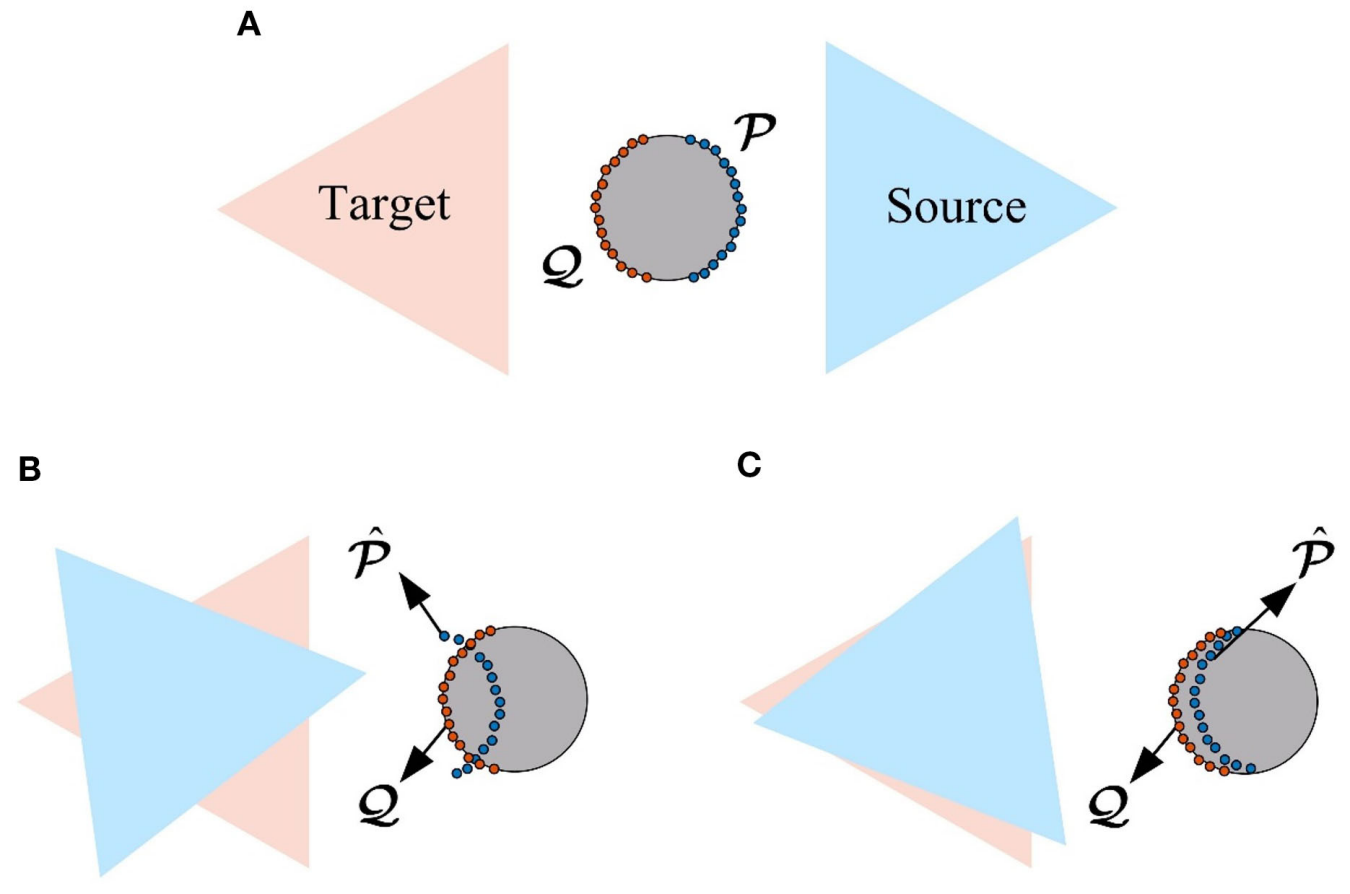
Over-registration illustration. **(A)** Normal registration (top view). **(B)** Excessive translation (top view). **(C)** Excessive rotation (top view).

To further improve the registration accuracy and quality, fine registration is performed using the point-to-point iterative closest point (ICP) algorithm (Besl and McKay, 1992), which minimizes the Euclidean distance between corresponding points to iteratively achieve optimal alignment. However, we observed that in practice, excessive pursuit of local minimization can lead to overfitting, a phenomenon we term “over-registration.” This issue may cause the final alignment deviating from the true geometric structure (Kelbe et al., 2016), as illustrated in **Figure 8**.

To address this issue and ensure the alignment is focused on the key structural features, our primary strategy is to exclude the ground and trunk point clouds from the ICP process, which are more prone to causing local minima convergence, and use only the remaining branch point clouds for registration. This is because these geometrically simple structures constitute a dominant portion of the total point cloud, and their high consistency between scans can dominate the registration’s error metric, masking misalignments in the more complex branching architecture. In contrast, the morphology of branches is more complex and diverse, making them better representations of local features from different viewpoints of the same tree. These features provide independent constraints, helping to prevent convergence to local minima, while enhancing registration stability and accuracy in low-overlap scenarios.

Additionally, to further refine this process and improve stability, we introduce the “theoretical overlap ratio” parameter from the CloudCompare core library. In our branch-only ICP process, we empirically set this value to 90%. This directs the algorithm to, in each iteration, effectively filter out the 10% of point-pair correspondences with the largest Euclidean distances. This supplementary measure further reduces the influence of potentially unreliable correspondences, ensuring the registration is robust against residual noise or minor misalignments. This two-step process ensures that the final alignment is determined by the most reliable correspondences on the tree’s branching structure, yielding a high-fidelity result. By using this ICP algorithm optimized for low-overlap cases, we obtain the transformation matrix $T_{f}$ for fine registration. This allows us to compute the overall rotation-translation matrix for point cloud registration as $T=T_{f}T_{c}$ . And then ultimately align the source and target point clouds according to Equation 3, completing the 3D reconstruction of the apple tree point cloud.

In summary, by optimizing the processing flow and strategies, the proposed method achieves marker-free, automated registration of low-overlap point clouds for individual apple trees. Designed specifically for low-overlap scenarios, this method utilizes local matching of the trunk and branches to effectively address the challenges of feature extraction and registration from different viewpoints, thereby completing the 3D reconstruction of point clouds.

### 3.4 Evaluation metrics

We tested our proposed registration algorithm on the Apple-Trees dataset and quantitatively evaluated the registration results using the ground truth. Based on the overall workflow and the intermediate values and results that may arise during the process, we use the following three evaluation metrics:

- Success rate (Wang X. et al., 2023). The overall success of our registration pipeline hinges on the coarse registration’s ability to establish the correct initial alignment. We therefore define a successful trial based on a direct measure of our core contribution: a registration is considered a “success” if the best matching branch identified by our BranchMatch algorithm matches the manually annotated ground-truth correspondence. This approach provides a meaningful evaluation of our method’s matching robustness. The final success rate is then calculated as the number of successful registrations divided by the total number of trials.
- Matrix-based errors (Yang et al., 2016). To evaluate the accuracy of the estimated transformation, we compare the transformation matrix obtained by our BranchMatch algorithm with the ground truth. As expressed in Equation 4, each transformation matrix can be decomposed into a rotation matrix R and a translation vector t . Based on this, we define two error metrics:
- Rotation error is computed using the axis-angle representation. Equation 8 gives the rotation angle β of a single rotation matrix R , while Equation 9 calculates the angular difference between the estimated rotation R and the ground truth rotation $\tilde{R}$ . Both are derived using Rodrigues’ formula:

(8) $$\beta =\arccos(\frac{\text{tr}(R)-1}{2})$$

(9) $$e_{R}=\arccos\begin{array}{c} \left(\frac{\text{tr}(\tilde{R}R^{T})-1}{2}\right) \end{array}$$

where tr(R) is the trace of matrix R .

- Translation error is defined as the Euclidean distance between the estimated translation vector t and the ground-truth translation $\tilde{t}$ (Equation 10):

(10) $$e_{t}=∥t-\tilde{t}∥$$

- Pointwise error (Chen et al., 2020). This metric reflects the registration accuracy at the point level. It is calculated as the average Euclidean distance between the transformed coordinates in our method and the coordinates transformed by the ground truth for all points in the source point cloud, as shown in Equation 11:

(11) $$e_{p}=\frac{1}{N_{p}^{*}}\sum_{i=1}^{N_{p}^{*}}∥(Rp_{i}^{*}+t)-(\tilde{R}p_{i}^{*}+\tilde{t})∥$$

where $N_{p}^{*}$ is the number of points in the source point cloud, and $p_{i}^{*}$ is the *i*-th point.

## 4 Results and discussion

Our algorithm was implemented in C++ using the Point Cloud Library (Rusu and Cousins, 2011) and CCCoreLib (Girardeau-Montaut, 2006). All operations were performed on a computer equipped with an Intel Core i5 CPU (16 threads) and 32 GB RAM.

### 4.1 Registration results

This study aims to register two 180°-separated, low-overlap point clouds. The algorithm is tested on the Apple-Trees dataset under these conditions. Due to its symmetry in source-target selection, registration results are analyzed in two configurations: S1 (target) with S4 (source) and S2 (target) with S3 (source).

#### 4.1.1 Coarse registration results

**Figure 9:**
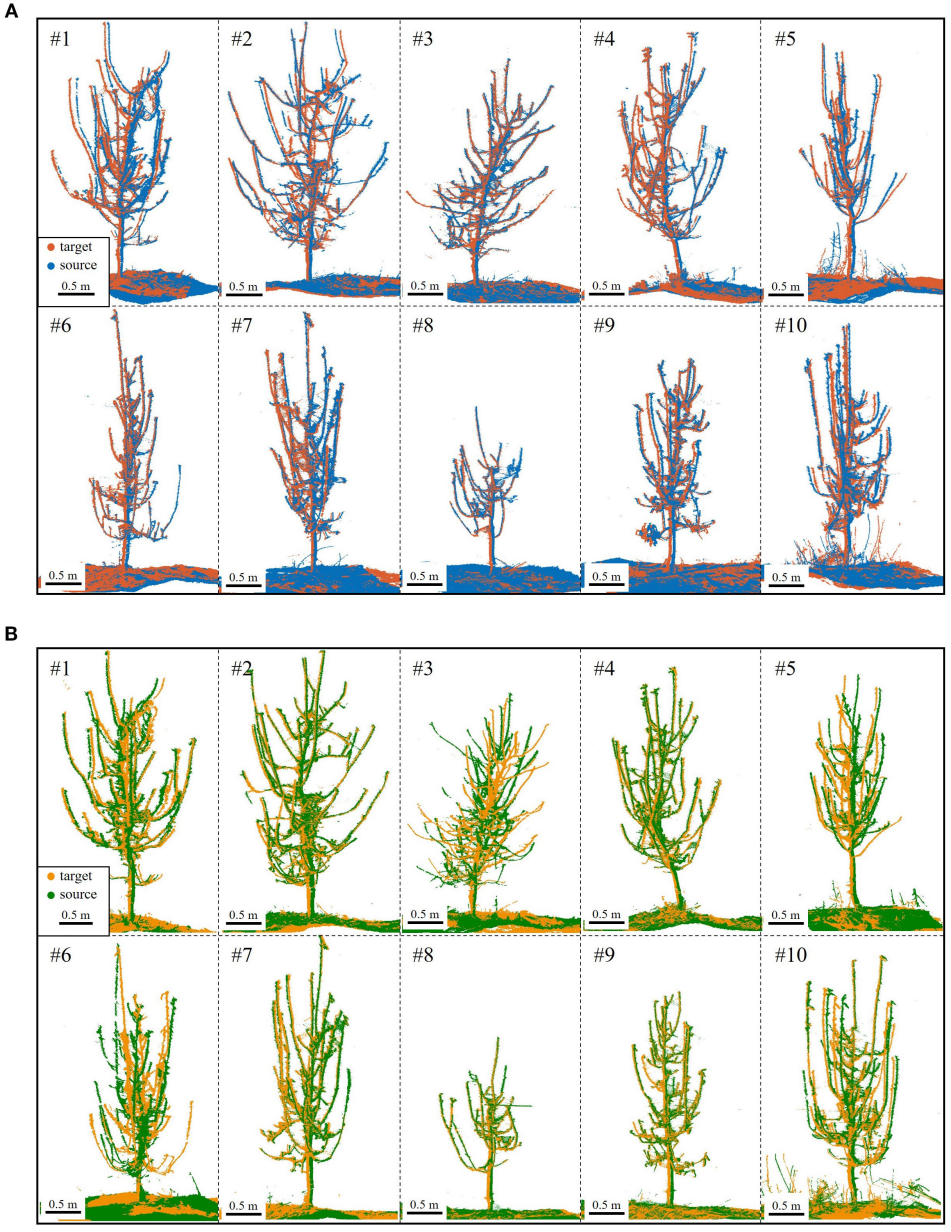
Coarse registration results. **(A)** S4 (blue) to S1 (red) for Trees #1-#10. **(B)** S3 (green) to S2 (yellow) for Trees #1-#10; registration failed for Tree #3 and Tree #6.

Coarse registration, a critical step, provides an initial alignment for fine registration. **Figure 9** shows the coarse registration results under S1-S4 and S2-S3 conditions. In S2-S3, Tree #3 and Tree #6 failed to match branches, resulting in an 18/20 success rate (90%).

**Figure 9** reveals that despite coarse registration, local misalignments remain, primarily as slight rotational deviations. However, the ground and trunk sections align almost perfectly, confirming their high overlap across viewpoints. In contrast, branches exhibit greater morphological variation, better capturing local features. As a result, visual errors are smaller in trees with thicker branches and clearer point clouds.

In addition to success rate, coarse registration requires accuracy. For the proposed method, rotation plays a decisive role (**Figure 9**), with rotational error indicating correct branch matching. Translation error, primarily from trunk cylinder center point inaccuracies due to ground and trunk fitting, has minimal impact. Pointwise error, reflecting distances between corresponding points, is a key metric for registration accuracy. Thus, **Table 2** summarizes rotational and pointwise errors for each tree under both alignment conditions.

**Table 2:** Statistics on coarse registration error.

Tree ID	*e*_*R*_ (mrad)		*e*_*p*_ (mm)	
	S1-S4	S2-S3	S1-S4	S2-S3
#1	143.58	131.44	54.07	53.13
#2	106.27	27.40	47.15	17.14
#3	24.53	–	15.45	–
#4	54.43	41.79	26.92	13.89
#5	221.32	469.19	57.47	113.47
#6	11.35	–	11.79	–
#7	81.94	48.10	29.56	18.05
#8	35.59	21.09	14.63	9.47
#9	106.74	18.20	28.86	6.30
#10	120.84	214.13	47.12	65.46
Average	104.33		35.00	

*e*_*R*_ represents the rotation error of matrix-based errors, while *e*_*p*_ denotes the pointwise error.

“-” denotes registration failure.

Analyzing the data, the coarse registration achieved an average rotation error of 104.33 mrad (~5.98°) and a pointwise error of 35.00 mm for 180°-separated viewpoints, satisfying ICP algorithm requirements. Tree #6 (S1-S4) and Tree #9 (S2-S3) exhibited the lowest errors, indicating the most accurate registration and aligning with visual results in **Figure 9**. In contrast, Tree #5 showed the highest errors in both configurations. Despite correct branch matching, its registration accuracy is lower due to the challenge of fitting its thin branches—as shown in **Table 1**, its maximum and average basal branch diameters are only 1.7 cm and 1.4 cm, respectively, the lowest in the dataset—leading to higher errors.

Moreover, a failure analysis of the S2-S3 configuration provides further insight into the algorithm’s mechanics. The two failures, Tree #3 and Tree #6, occurred for different, viewpoint-dependent reasons. For Tree #3, its failure can be attributed to its extreme structural complexity; with the highest branch count (18), the S2-S3 perspective resulted in severe inter-branch occlusion. For Tree #6, the challenge was structural ambiguity; several of its primary branches had highly similar geometric features, a result of deviating from standard pruning guidelines. This ambiguity, combined with its small crown width, created a geometrically confusing profile from the S2-S3 perspective, leading to matching failure. Crucially, the success of both trees in the S1-S4 configuration validates our design philosophy. It demonstrates that our algorithm’s success is not dependent on overlap percentage, but on its ability to leverage the holistic architectural information provided by a given viewpoint. When a perspective like S1-S4 provides clear, unambiguous structural cues—even for a dense or atypical tree—BranchMatch can robustly and efficiently succeed under challenging low-overlap conditions.

#### 4.1.2 Fine registration results

**Figure 10:**
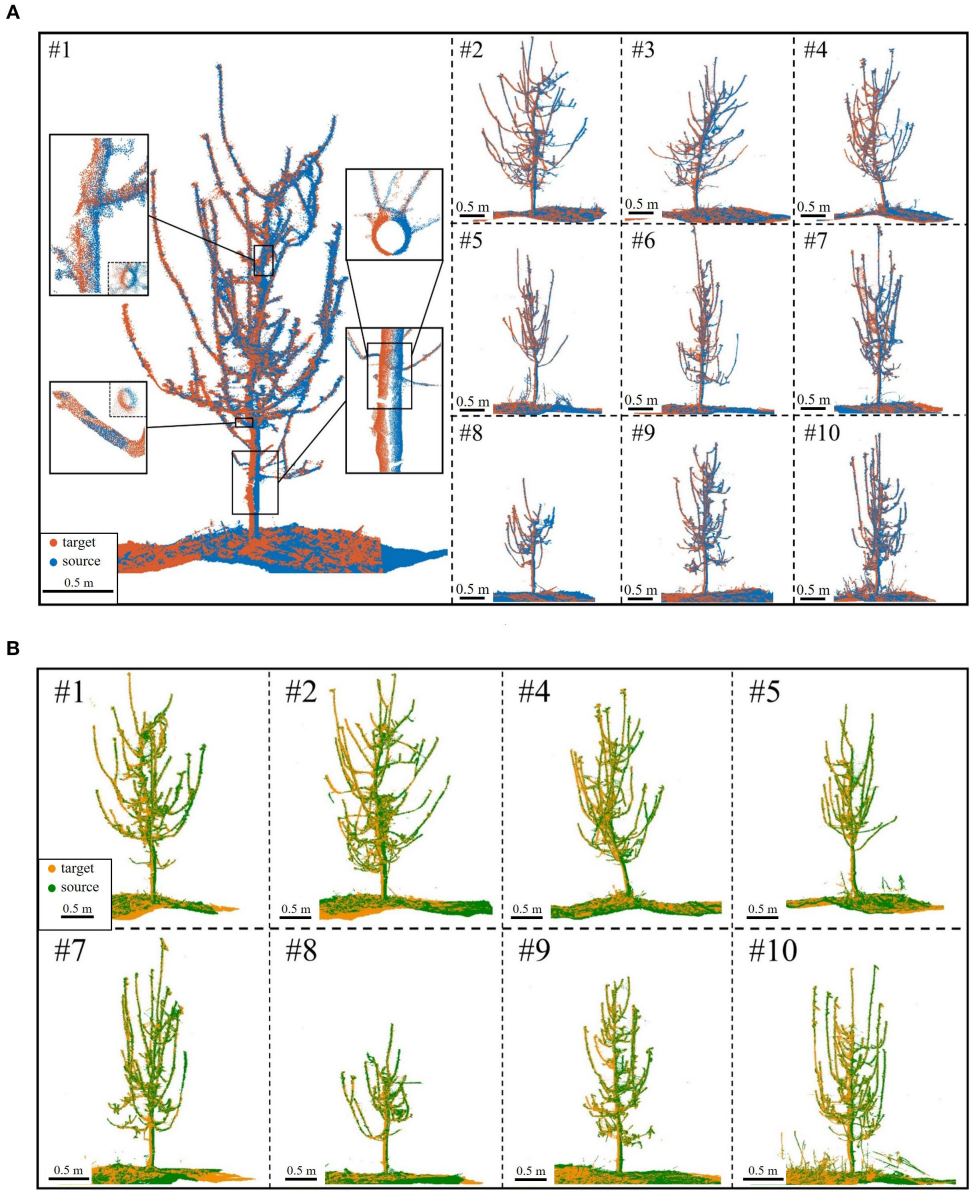
Fine registration results. **(A)** S4 (blue) to S1 (red) for Trees #1-#10. **(B)** S3 (green) to S2 (yellow) for Trees #1-#10; registration failed for Tree #3 and Tree #6.

Following coarse registration, ICP-based optimization was applied for branch-focused fine registration. **Figure 10** shows the results: missing point clouds from single viewpoints were filled, revealing finer tree details, with remaining gaps due to external occlusions like drip irrigation tapes. A local zoom-in of side and top views at various heights shows a near “perfectly halved” distribution in low-overlap regions. This confirms that the proposed method effectively mitigates over-registration while achieving high-accuracy alignment.

Beyond visual assessment, quantifying fine registration errors is essential. Matrix-based errors evaluate rotation and translation performance, while pointwise errors measure Euclidean distance discrepancies between corresponding points. **Table 3** presents fine registration errors for both S1-S4 and S2-S3 conditions.

**Table 3:** Statistics on fine registration error.

Case	Tree ID	Matrix-based errors		Pointwise errors			
		*e*_*R*_ (mrad)	*e*_*t*_ (mm)	*e*_*p*_-x (mm)	*e*_*p*_-y (mm)	*e*_*p*_-z (mm)	*e*_*p*_ (mm)
S1-S4	#1	0.97	3.53	2.08	1.37	0.22	2.52
	#2	1.74	3.93	2.26	0.45	0.44	2.38
	#3	1.30	3.11	2.56	0.32	0.27	2.62
	#4	3.58	6.08	3.21	1.71	1.61	4.14
	#5	1.65	4.77	1.35	0.43	1.21	1.90
	#6	1.74	3.13	0.67	1.42	1.61	2.41
	#7	1.31	3.84	1.55	1.12	0.20	2.01
	#8	1.30	1.15	2.24	0.80	1.10	2.65
	#9	2.68	6.04	1.78	0.47	0.55	2.06
	#10	1.48	4.64	1.30	0.96	2.52	3.04
S2-S3	#1	1.54	3.30	1.90	1.69	0.40	2.59
	#2	3.00	7.94	2.99	2.64	1.00	4.20
	#3	–	–	–	–	–	–
	#4	1.54	1.46	0.63	1.46	1.20	2.09
	#5	0.86	3.85	3.23	0.45	0.59	3.33
	#6	–	–	–	–	–	–
	#7	2.43	6.44	0.89	1.41	1.82	2.69
	#8	1.37	2.84	0.20	0.44	0.95	1.11
	#9	3.27	7.30	2.83	1.85	0.78	3.55
	#10	2.90	4.53	1.05	2.09	2.04	3.35
Average		1.93	4.33	1.82	1.17	1.03	2.70

*e*_*R*_ and *e*_*t*_ represent the rotation and translation errors, respectively. *e*_*p*_-x, *e*_*p*_-y, and *e*_*p*_-z represent the pointwise error in each direction, i.e., the error in the coordinates of the points along the x, y, and z axes. while *e*_*p*_ denotes the pointwise error.

“-” denotes registration failure.

As shown in **Table 3**, the algorithm achieves high accuracy in registering low-overlap apple tree point clouds 180° apart. The average rotation error is just 1.93 mrad (~0.11°), ensuring precise alignment without distortion or significant misalignment. The average translation error is 4.33 mm, demonstrating millimeter-level precision despite orchard complexities and intricate branching. The average pointwise error is 2.70 mm, with errors of 1.82 mm, 1.17 mm, and 1.03 mm in the X, Y, and Z directions, respectively, indicating a uniform distribution without significant bias. Notably, the pointwise error magnitude reflects local alignment quality. In complex branch structures, the reduced pointwise error (variance: 0.59 mm) indicates the algorithm’s strong adaptability to high-complexity scenarios.

Further analysis of individual tree registration results reveals that Tree #2 during the S2-S3 alignment exhibits the highest pointwise error, reaching 4.20 mm, significantly above average. This is due to its intricate branching and low-overlap affecting alignment accuracy. Nevertheless, as shown in **Figure 10B**, the overall error remains within an acceptable range. Visual assessment confirms well-aligned global structure and local details, with no significant geometric distortions, demonstrating the algorithm’s robustness in error control under complex conditions. In simpler scenarios, accuracy improves further. For example, Tree #8 achieves a pointwise error of just 1.11 mm in the S2-S3 alignment, demonstrating the algorithm’s superior performance when registering trees with distinct features in less complex environments.

The results confirm the effectiveness and robustness of the proposed algorithm in handling complex, low-overlap point clouds. Error analysis highlights its capability to manage challenges from intricate structures and local occlusions, demonstrating its potential to replace traditional marker-based methods. Overall, the algorithm achieves high-precision two-station registration for apple tree point clouds, enabling 3D reconstruction.

#### 4.1.3 Evaluation of reconstructed model utility

To quantitatively evaluate the practical utility of the 3D models generated by our two-station method, we compared them against the more complete four-station ground-truth reconstructions. The data for both models underwent identical preprocessing steps before we assessed both the geometric completeness and the accuracy of key extracted structural parameters. For the completeness metric, we used a point-to-point nearest neighbor distance; a point in the ground-truth model was considered “covered” if its nearest neighbor in the two-station model was within a 5 mm threshold. This threshold was chosen as a reasonable tolerance, as it is slightly larger than the scanner’s instrumental accuracy (up to 2 mm) and our method’s average pointwise registration error (2.70 mm). For the accuracy of the key structural parameters, we assessed the Absolute Error (AE) for each individual sample, calculated against its ground-truth value.

The results, summarized in **Table 4**, confirm that our efficient approach yields high-fidelity models suitable for subsequent phenotyping tasks. Specifically, in terms of completeness, the two-station models achieved an average coverage rate of 95.6%, indicating that the vast majority of the tree’s structure is successfully captured. Furthermore, the accuracy of the extracted parameters is exceptionally high. The Mean Absolute Error (MAE) for tree height was only 0.03 cm (RMSE 0.04 cm), for basal trunk diameter it was 0.11 cm (RMSE 0.14 cm), and for the maximum primary branch diameter, the MAE was just 0.17 cm (RMSE 0.18 cm). The close agreement between the MAE and RMSE values is particularly noteworthy, as it indicates a uniform error distribution without significant outliers, which speaks to the stability and reliability of the proposed method.

**Table 4:** Comparison of key structural parameters extracted from the two-station model vs. the four-station ground-truth model.

Tree ID	Completeness (%)		AE. Tree height (cm)		AE. Basal Trunk Diameter (cm)		AE. Max. Branch Diameter (cm)	
	S1-S4	S2-S3	S1-S4	S2-S3	S1-S4	S2-S3	S1-S4	S2-S3
#1	95.88	96.59	0.02	0.05	0.16	0.18	0.21	0.20
#2	94.87	96.29	0.06	0.03	0.10	0.25	0.27	0.23
#3	94.76	–	0.07	–	0.09	–	0.12	–
#4	96.14	95.09	0.04	0.01	0.13	0.20	0.15	0.11
#5	98.38	96.76	0.04	0.05	0.28	0.01	0.06	0.31
#6	92.97	–	0.05	–	0.06	–	0.11	–
#7	95.63	96.58	0.01	0.05	0.08	0.06	0.24	0.11
#8	95.36	96.86	0.02	0.03	0.03	0.04	0.13	0.05
#9	96.01	93.22	0.03	0.04	0.11	0.18	0.18	0.16
#10	94.69	94.31	0.04	0.03	0.08	0.07	0.19	0.21
Avg	95.58		0.03		0.11		0.17	

AE stands for Absolute Error, calculated as the absolute difference between the two-station and four-station model values. The “Avg” row represents the Mean Absolute Error (MAE) for structural parameters of all successful registrations. “-” denotes registration failure.

These results provide strong quantitative evidence that the models produced by our proposed method successfully balance acquisition efficiency with reconstruction accuracy, making them highly valuable for practical applications in precision agriculture.

### 4.2 Comparison with alternative strategies and methods

To comprehensively validate the effectiveness of low-overlap point cloud registration for two stations 180° apart, we compare its results with those of high-overlap multi-station registration and other registration algorithms.

#### 4.2.1 High-overlap multi-station registration

**Figure 11:**
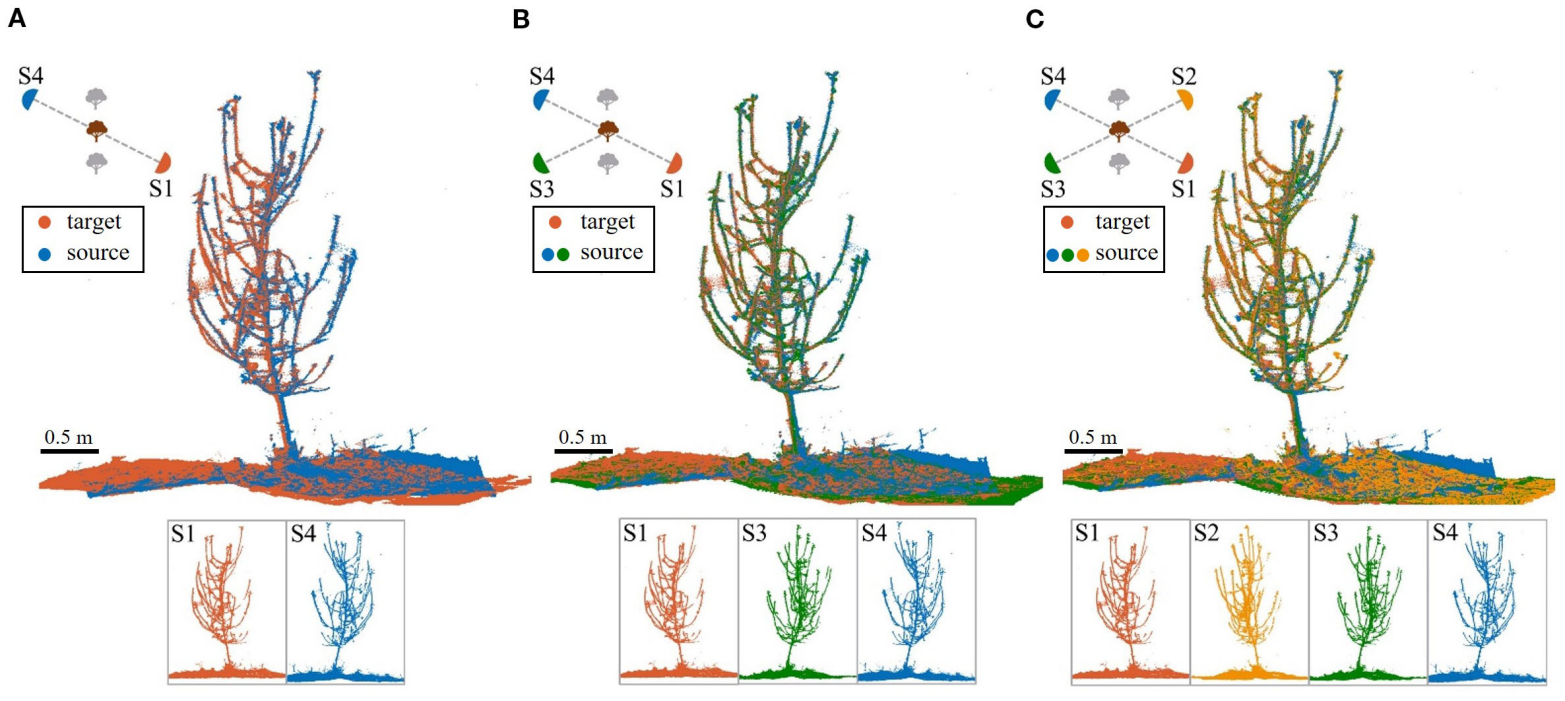
Registration results for Tree #4. **(A)** Two-station. **(B)** Three-station. **(C)** Four-station.

For multi-station high-overlap registration, we employ a cumulative registration strategy. As shown in **Figure 11**, for three-station registration, using S1 as the target, S3 and S4 are sequentially aligned: first, S3 to S1; then, S4 to the combined S1+S3’ (transformed S3). Similarly, four-station registration aligns S2-S3-S4 to S1 sequentially. This strategy starts with small-angle viewpoints and progressively accumulates aligned point clouds, ensuring high overlap.

Tree #4 is used as an example to visually compare the results of two-, three-, and four-station registrations (**Figure 11**). While multi-station registration slightly reduces errors and improves point cloud completeness, the improvement is minimal. In practical orchard settings, the two-station registration already captures nearly all parts of the tree, including detailed features, and is sufficient for various subsequent analyses.

Additionally, a comparison is made between two-station registration and cumulative registration with three- and four-station for all 10 trees in the dataset. **Table 5** presents the recorded results for three key alignment quality metrics, along with the average total time required for the entire process (including all registration steps):

**Table 5:** Comparison of two-station low overlap registration and multi-station high overlap registration results.

Experiment setup	Success rate	Avg. matrix-based errors		Avg. pointwise error	Avg. time
		*e*_*R*_ (mrad)	*e*_*t*_ (mm)	*e*_*p*_ (mm)	t (s)
two-station	90.00%	1.93	4.33	2.70	165.18
three- station	90.00%	1.72	3.76	2.29	380.04
four-station	80.00%	2.07	4.64	2.31	586.91

When calculating the average values for all metrics, failed cases are excluded. *e*_*R*_, *e*_*t*_, and *e*_*p*_ represent the average rotation error, translation error, and pointwise error, respectively.

As shown in **Table 5**, three- and four-station registrations reduce pointwise errors by only 0.41 mm and 0.39 mm compared to two-station registration. The success rate remains unchanged for the three-station setup, while it even decreases for the four-station configuration. Meanwhile, registration time nearly doubles, highlighting the significant computational complexity and time cost escalation with increasing input point clouds.

In theory, adding stations with smaller angular differences can increase local overlap and substantially improve registration performance. However, in our experiments, such improvements were minimal. This is primarily due to the robustness of the proposed BranchMatch algorithm under low-overlap conditions. By leveraging structurally stable features—particularly the trunk and key branch segments—the method achieves reliable matching even with two diametrically opposed scans, enabling accurate transformation estimation. Under such conditions, the structural benefits gained from additional viewpoints are significantly reduced, which further demonstrates the efficiency of the two-station strategy. In addition, further analysis shows that multi-station registration, while benefiting from higher overlap, introduces cumulative errors. As the number of stations increases, early-stage errors are amplified, potentially disrupting branch matching and lowering the overall success rate. In this study, errors and failures were most frequently observed at the final station, further highlighting the negative impact of cumulative errors. Therefore, while multi-station registration improves point cloud completeness, its accuracy gains are limited or even diminished, with increased computational costs and reduced robustness.

In summary, the proposed method achieves high success rates by matching structural features, even with high-overlap data, demonstrating adaptability to varying overlap conditions. Besides, given the 90% success rate and higher time costs of multi-station registration, two-station registration offers a better efficiency-accuracy balance.

#### 4.2.2 Other registration algorithms

To further validate the performance of our method, we conducted a direct comparison with several state-of-the-art (SOTA) registration algorithms on our Apple-Trees dataset. This was necessary due to the lack of publicly available point cloud datasets of trees captured from opposing, low-overlap viewpoints. We selected two representative deep learning-based methods, GCNet (a SOTA method on the 3DMatch dataset) (Zhu et al., 2022) and GeoTransformer (a SOTA method on the 3DLoMatch dataset) (Qin et al., 2022), as well as a robust traditional baseline, SAC-IA + ICP (using a standard ICP algorithm) (Rusu et al., 2009).

For GCNet, we used the official 3DMatch pre-trained model. The model employs a 4-layer KPConv encoder. It was trained for 40 epochs with a batch size of 1, using a voxel downsampling size of 1 cm and 256 sampled key points per fragment. A multi-stage GNN with 256-dimensional intermediate features and a cross-attention module is used to perform fine-grained correspondence estimation. For GeoTransformer, we also used its official 3DMatch pre-trained model. This model utilizes a 4-stage KPConv-FPN backbone with a 2.5 cm voxel size to extract 256-dimensional superpoint features. It was trained for 40 epochs using an Adam optimizer with an exponential decay learning rate schedule (γ = 0.05). GeoTransformer performs coarse-to-fine matching via a global transformer with geometry-invariant structural embeddings, followed by local refinement. As a traditional baseline, we implemented SAC-IA + ICP. For this method, the point cloud was downsampled with a 1 cm voxel size, normals were estimated with a 2 cm radius, and the FPFH feature radius was set to 5 cm. All methods used the same preprocessed data to ensure a fair comparison, and the results are summarized in **Table 6**.

**Table 6:** Comparison with SOTA registration methods on the Apple-Trees dataset.

Method	Avg. matrix-based errors		Avg. pointwise error
	*e*_*R*_ (mrad)	*e*_*t*_ (mm)	*e*_*p*_ (mm)
GCNet	21.77	48.17	20.05
GeoTransformer	9.54	18.63	7.48
SAC-IA + ICP(original)	9.50	27.65	16.78
BranchMatch(ours)	**1.93**	**4.33**	**2.70**

Bold values denote the best result among all compared methods for the corresponding metric.

The results clearly demonstrate the superior accuracy of BranchMatch in the specific domain of single-tree, low-overlap registration. The deep learning methods, particularly GCNet which is trained primarily on high-overlap indoor scenes, exhibited the largest errors. GeoTransformer, which includes optimizations for low-overlap scenarios, performed best among the learning-based approaches. The traditional SAC-IA method, while not requiring large-scale training data, still showed a significant accuracy gap compared to BranchMatch. This performance gap can be attributed to a fundamental difference in approach that BranchMatch leverages domain-specific structural knowledge, whereas the SOTA methods are general-purpose feature matchers.

The deep learning methods, trained on generic datasets, and SAC-IA, which relies on local FPFH features, both struggle in this challenging low-overlap scenario. This is because the severe viewpoint change between 180° scans results in very few repeatable local geometric patterns, and the inherent self-similarity of branches creates significant matching ambiguity for these general-purpose descriptors. This underscores the effectiveness of our structure-based feature matching strategy in achieving high-precision alignment where other general-purpose methods struggle.

What’s more, due to the limitation of parameter sensitivity, we also compare our results directly with those SOTA methods target the tree point cloud registration methods reported in existing literature.

Ma et al. (2021) developed a 3D reconstruction platform using two depth cameras, generating high-quality single-view point clouds based on recorded poses. Their automatic registration method, leveraging global skeleton extraction, achieved sub-1 mm pointwise errors for 55°-separated point clouds across various weather conditions. However, multi-station data were still required for complete tree reconstruction. For 180°-separated point clouds, their method achieved an average error of 2.61 mm (variance: 4.40 mm), while our method achieved 2.70 mm (variance: 0.59 mm), demonstrating superior robustness. Unlike their global skeleton extraction, BranchMatch focuses on key segment extraction, improving efficiency and accuracy. This design excels in complex branching scenarios, avoiding global extraction errors and enhancing robustness. Additionally, BranchMatch requires no specialized equipment or positional data, offering greater flexibility in data collection.

We further compared our method with the forest plot-scale registration approach of Wang X. et al. (2023) to highlight the different design trade-offs for distinct applications. Their stem-map-based method is optimized for plot-scale efficiency, achieving high computational speed with a corresponding accuracy of approximately 10 mm, which is well-suited for large-area inventory. In contrast, our BranchMatch algorithm is tailored for single-tree fidelity, intentionally prioritizing millimeter-level accuracy (~2.7 mm) for precision tasks like robotic pruning, where per-tree detail is critical. Furthermore, our method’s demonstrated success in registering point clouds with low overlap and in complex branching scenarios significantly expands the applicability of high-precision, single-tree registration, providing the superior accuracy and adaptability required for advanced precision applications.

### 4.3 Limitations

A potential limitation, common to registration methods that rely on fitting geometric primitives to real-world data, is their theoretical susceptibility to error propagation. In our approach, for instance, minor viewpoint-dependent variations—such as those introduced by topographic inconsistencies—may result in small discrepancies in the estimated ground plane across scans. These initial deviations can propagate through subsequent steps, potentially affecting the localization of trunks and key branches. Such propagation may influence feature-based thresholding, thereby increasing the risk of branch matching failures under certain conditions.

Additionally, the BranchMatch method employs several empirical thresholds (e.g., for cylindrical fitting and branch features) tailored to the standardized architectural properties of tall-spindle apple trees. Although these values were determined through data-informed tuning and conservatively selected to accommodate noise and natural variation, their optimality may still vary with different data quality or sensor characteristics. This represents a common limitation of parameter-dependent methods, and future work could thus explore adaptive thresholding strategies to enhance the method’s robustness and generalizability across diverse scenarios.

## 5 Conclusion

This paper introduces BranchMatch, a high-precision and rapid 3D point cloud reconstruction method tailored for individual tall-spindle apple trees during their dormant period. The method focuses on marker-free point cloud registration in low-overlap scenarios. The core of this research lies in the accurate identification and utilization of critical geometric structural features of the apple tree. By employing a structure features-based strategy and incorporating a dynamic weighted feature discrimination function, BranchMatch enables precise matching of key point cloud features, even in cases of complex tree trunks and branches with low overlap. Furthermore, an ICP algorithm optimized for low-overlap registration enhances both efficiency and accuracy, preventing over-registration and further boosting the method’s robustness.

Experimental results demonstrate the outstanding performance of BranchMatch in terms of both accuracy and efficiency. Compared to traditional marker-based registration methods, BranchMatch achieves an average pointwise error of 2.70 mm with a variance of 0.59 mm. In terms of computational efficiency, the method only requires two point clouds captured 180° apart to reconstruct a nearly complete tree model, outperforming traditional multi-view, high-overlap registration methods. This makes it highly suitable for large-scale orchard applications. Testing on the Apple-Trees dataset further confirms the method’s adaptability and robustness, laying a strong foundation for the future deployment of automated orchard management systems.

In conclusion, BranchMatch provides a promising solution for accurate 3D modeling of dormant, tall-spindle apple trees in complex agricultural environments. By reducing the number of measurement stations and overcoming the challenges posed by low-overlap views, the method significantly improves data acquisition and processing efficiency. Future work will focus on broadening the applicability of the proposed method to include registration tasks involving multiple types of individual fruit trees, entire orchard-scale or forest-plots datasets, assessing its robustness across a wider range of conditions, and validating its adaptability across data acquired from sensors with different precision levels. Additionally, when integrated with dynamic orchard management monitoring, this approach could provide extensive data support and technical solutions for smart agriculture and forestry.

## Data Availability

The datasets presented in this study can be found in online repositories. The names of the repository/repositories and accession number(s) can be found below: https://github.com/NingWang1999/BranchMatch.

## References

[B1] Besl P. J. McKay N. D. (1992). A method for registration of 3-d shapes. IEEE Trans. Pattern Anal. Mach. Intell. 14, 239–256. doi: 10.1109/34.121791

[B2] Bucksch A. Khoshelham K. (2013). Localized registration of point clouds of botanic trees. IEEE Geosci. Remote Sens. Lett. 10, 631–635. doi: 10.1109/LGRS.2012.2216251

[B3] Cai Z. Chin T. Bustos A. P. Schindler K. (2019). Practical optimal registration of terrestrial lidar scan pairs. Isprs-J. Photogramm. Remote Sens. 147, 118–131. doi: 10.1016/j.isprsjprs.2018.11.016

[B4] Calders K. Adams J. Armston J. Bartholomeus H. Bauwens S. Bentley L. P.. (2020). Terrestrial laser scanning in forest ecology: expanding the horizon. Remote Sens. Environ. 251, 112102. doi: 10.1016/j.rse.2020.112102

[B5] Chattopadhyay S. Akbar S. A. Elfiky N. M. Medeiros H. Kak A. (2016). “Measuring and modeling apple trees using time-of-flight data for automation of dormant pruning applications,” in 2016 IEEE Winter Conference on Applications of Computer Vision (WACV) (Lake Placid, NY, USA), 1–9. doi: 10.1109/WACV.2016.7477596

[B6] Chen S. Nan L. Xia R. Zhao J. Wonka P. (2020). Plade: a plane-based descriptor for point cloud registration with small overlap. IEEE Trans. Geosci. Remote Sens. 58, 2530–2540. doi: 10.1109/TGRS.2019.2952086

[B7] Cheng L. Chen S. Liu X. Xu H. Wu Y. Li M.. (2018). Registration of laser scanning point clouds: a review. Sensors 18, 1641. doi: 10.3390/s18051641, PMID: 29883397 PMC5981425

[B8] Cheng X. Liu X. Li J. Zhou W. (2024). Deep learning-based point cloud registration: a comprehensive investigation. Int. J. Remote Sens. 45, 3412–3442. doi: 10.1080/01431161.2024.2343434

[B9] Dai W. Kan H. Tan R. Yang B. Guan Q. Zhu N.. (2022). Multisource forest point cloud registration with semantic-guided keypoints and robust ransac mechanisms. Int. J. Appl. Earth Obs. Geoinf. 115, 103105. doi: 10.1016/j.jag.2022.103105

[B10] Dassot M. Constant T. Fournier M. (2011). The use of terrestrial lidar technology in forest science: application fields, benefits and challenges. Ann. For. Sci. 68, 959–974. doi: 10.1007/s13595-011-0102-2

[B11] Deng Z. Yao Y. Deng B. Zhang J. (2021). “A robust loss for point cloud registration,” in Proceedings of the IEEE/CVF International Conference on Computer Vision (ICCV) (Montreal, QC, Canada: IEEE), 6118–6127. doi: 10.1109/ICCV48922.2021.00608

[B12] Dong Z. Liang F. Yang B. Xu Y. Zang Y. Li J.. (2020). Registration of large-scale terrestrial laser scanner point clouds: a review and benchmark. Isprs-J. Photogramm. Remote Sens. 163, 327–342. doi: 10.1016/j.isprsjprs.2020.03.013

[B13] Fischler M. A. Bolles R. C. (1981). Random sample consensus: a paradigm for model fitting with applications to image analysis and automated cartography. Commun. ACM 24, 381–395. doi: 10.1145/358669.358692

[B14] Fu Y. Xia Y. Zhang H. Fu M. Wang Y. Fu W.. (2023). Skeleton extraction and pruning point identification of jujube tree for dormant pruning using space colonization algorithm. Front. Plant Sci. 13, 1103794. doi: 10.3389/fpls.2022.1103794, PMID: 36743548 PMC9893002

[B15] Girardeau-Montaut D. (2006). Détection de Changement sur des Données géométriques Tridimensionnelles. Doctoral Dissertation, Télécom ParisTech, Palaiseau, France.

[B16] Guan H. Su Y. Sun X. Xu G. Li W. Ma Q.. (2020). A marker-free method for registering multi-scan terrestrial laser scanning data in forest environments. Isprs-J. Photogramm. Remote Sens. 166, 82–94. doi: 10.1016/j.isprsjprs.2020.06.002

[B17] He L. Schupp J. (2018). Sensing and automation in pruning of apple trees: a review. Agronomy 8, 211. doi: 10.3390/agronomy8100211

[B18] Henning J. G. Radtke P. J. (2008). Multiview range-image registration for forested scenes using explicitly-matched tie points estimated from natural surfaces. Isprs-J. Photogramm. Remote Sens. 63, 68–83. doi: 10.1016/j.isprsjprs.2007.07.006

[B19] Ho S. Nieto L. G. Rickard B. J. Reig G. Lordan J. Lawrence B. T.. (2024). Effects of cultivar, planting density and rootstock on long-term economic performance of apple orchards in the northeastern u.s. Sci. Hortic. 332, 113194. doi: 10.1016/j.scienta.2024.113194

[B20] Karkee M. Adhikari B. Amatya S. Zhang Q. (2014). Identification of pruning branches in tall spindle apple trees for automated pruning. Comput. Electron. Agric. 103, 127–135. doi: 10.1016/j.compag.2014.02.013

[B21] Kelbe D. van Aardt J. Romanczyk P. van Leeuwen M. Cawse-Nicholson K. (2016). Marker-free registration of forest terrestrial laser scanner data pairs with embedded confidence metrics. IEEE Trans. Geosci. Remote Sens. 54, 4314–4330. doi: 10.1109/TGRS.2016.2539219

[B22] Kolmanič S. Strnad D. Kohek Š. Benes B. Hirst P. Žalik B. (2021). An algorithm for automatic dormant tree pruning. Appl. Soft Comput. 99, 106931. doi: 10.1016/j.asoc.2020.106931

[B23] Li J. Wu H. Xiao Z. Lu H. (2022). 3d modeling of laser-scanned trees based on skeleton refined extraction. Int. J. Appl. Earth Obs. Geoinf. 112, 102943. doi: 10.1016/j.jag.2022.102943

[B24] Liang X. Hyyppä J. Kaartinen H. Lehtomäki M. Pyörälä J. Pfeifer N.. (2018). International benchmarking of terrestrial laser scanning approaches for forest inventories. Isprs-J. Photogramm. Remote Sens. 144, 137–179. doi: 10.1016/j.isprsjprs.2018.06.021

[B25] Liang X. Kankare V. Hyyppä J. Wang Y. Kukko A. Haggrén H.. (2016). Terrestrial laser scanning in forest inventories. Isprs-J. Photogramm. Remote Sens. 115, 63–77. doi: 10.1016/j.isprsjprs.2016.01.006

[B26] Liu J. Liang X. Hyyppä J. Yu X. Lehtomäki M. Pyörälä J.. (2017). Automated matching of multiple terrestrial laser scans for stem mapping without the use of artificial references. Int. J. Appl. Earth Obs. Geoinf. 56, 13–23. doi: 10.1016/j.jag.2016.11.003

[B27] Lv X. Zhang X. Gao H. He T. Lv Z. Zhangzhong L. (2024). When crops meet machine vision: a review and development framework for a low-cost nondestructive online monitoring technology in agricultural production. Agric. Commun. 2, 100029. doi: 10.1016/j.agrcom.2024.100029

[B28] Ma B. Du J. Wang L. Jiang H. Zhou M. (2021). Automatic branch detection of jujube trees based on 3d reconstruction for dormant pruning using the deep learning-based method. Comput. Electron. Agric. 190, 106484. doi: 10.1016/j.compag.2021.106484

[B29] Martínez-Otzeta J. M. Rodríguez-Moreno I. Mendialdua I. Sierra B. (2023). Ransac for robotic applications: a survey. Sensors 23 (1), 327. doi: 10.3390/s23010327, PMID: 36616922 PMC9824669

[B30] Miao Y. Li S. Wang L. Li H. Qiu R. Zhang M. (2023). A single plant segmentation method of maize point cloud based on euclidean clustering and k-means clustering. Comput. Electron. Agric. 210, 107951. doi: 10.1016/j.compag.2023.107951

[B31] Miller J. Morgenroth J. Gomez C. (2015). 3d modelling of individual trees using a handheld camera: accuracy of height, diameter and volume estimates. Urban For. Urban Green. 14, 932–940. doi: 10.1016/j.ufug.2015.09.001

[B32] Monji-Azad S. Hesser J. Löw N. (2023). A review of non-rigid transformations and learning-based 3d point cloud registration methods. Isprs-J. Photogramm. Remote Sens. 196, 58–72. doi: 10.1016/j.isprsjprs.2022.12.023

[B33] Peng Y. Lin S. Wu H. Cao G. (2023). Point cloud registration based on fast point feature histogram descriptors for 3d reconstruction of trees. Remote Sens. 15 (15), 3775. doi: 10.3390/rs15153775

[B34] Pfeifer N. Gorte B. Winterhalder D. (2004). “Automatic reconstruction of single trees from terrestrial laser scanner data.” In: Proceedings of the 20th ISPRS Congress (Princeton, NJ, USA: Citeseer), 114–119.

[B35] Qin Z. Yu H. Wang C. Guo Y. Peng Y. Xu K.. (2022). “Geometric transformer for fast and robust point cloud registration,” In: Proceedings of the IEEE/CVF Conference on Computer Vision and Pattern Recognition (CVPR) (New Orleans, LA, USA: IEEE), 11163–11172. doi: 10.1109/CVPR52688.2022.01086

[B36] Raumonen P. Kaasalainen M. Åkerblom M. Kaasalainen S. Kaartinen H. Vastaranta M.. (2013). Fast automatic precision tree models from terrestrial laser scanner data. Remote Sens. 5, 491–520. doi: 10.3390/rs5020491

[B37] Robinson T. L. Hoying S. A. Reginato G. H. (2006). The tall spindle apple production system. New York Fruit Q. 14, 21–28. doi: 10.17660/ActaHortic.2011.903.79

[B38] Rusu R. B. Blodow N. Beetz M. (2009). “Fast point feature histograms (FPFH) for 3D registration,” in Proceedings of the 2009 IEEE International Conference on Robotics and Automation (ICRA) (Kobe, Japan: IEEE), 3212–3217. doi: 10.1109/ROBOT.2009.5152473

[B39] Rusu R. B. Cousins S. (2011). “3d is here: point cloud library (pcl),” in Proceedings of the 2011 IEEE International Conference on Robotics and Automation (ICRA) (Shanghai, China: IEEE), 1–4. doi: 10.1109/ICRA.2011.5980567

[B40] Salehi B. Jarahizadeh S. Sarafraz A. (2022). An improved ransac outlier rejection method for uav-derived point cloud. Remote Sens. 14 (19), 4917. doi: 10.3390/rs14194917

[B41] Shao J. Zhang W. Mellado N. Wang N. Jin S. Cai S.. (2020). Slam-aided forest plot mapping combining terrestrial and mobile laser scanning. Isprs-J. Photogramm. Remote Sens. 163, 214–230. doi: 10.1016/j.isprsjprs.2020.03.008

[B42] Shimizu K. Nishizono T. Kitahara F. Fukumoto K. Saito H. (2022). Integrating terrestrial laser scanning and unmanned aerial vehicle photogrammetry to estimate individual tree attributes in managed coniferous forests in Japan. Int. J. Appl. Earth Obs. Geoinf. 106, 102658. doi: 10.1016/j.jag.2021.102658

[B43] Sun S. Li C. Chee P. W. Paterson A. H. Meng C. Zhang J.. (2021). High resolution 3d terrestrial lidar for cotton plant main stalk and node detection. Comput. Electron. Agric. 187, 106276. doi: 10.1016/j.compag.2021.106276

[B44] Vasylieva N. Harvey J. (2021). Production and trade patterns in the world apple market. Innovative Marketing 17, 16. doi: 10.21511/im.17(1).2021.02

[B45] Wang J. Liu T. (2022). Spatiotemporal evolution and suitability of apple production in China from climate change and land use transfer perspectives. Food Energy Secur. 11, e386. doi: 10.1002/fes3.386

[B46] Wang L. Miao Y. Han Y. Li H. Zhang M. Peng C. (2023). Extraction of 3d distribution of potato plant cwsi based on thermal infrared image and binocular stereovision system. Front. Plant Sci. 13, 1104390. doi: 10.3389/fpls.2022.1104390, PMID: 36762177 PMC9903339

[B47] Wang X. Yang Z. Cheng X. Stoter J. Xu W. Wu Z.. (2023). Globalmatch: registration of forest terrestrial point clouds by global matching of relative stem positions. Isprs-J. Photogramm. Remote Sens. 197, 71–86. doi: 10.1016/j.isprsjprs.2023.01.013

[B48] Wang Y. Zhou P. Geng G. An L. Zhang Q. (2024). Low-overlap point cloud registration with transformer. IEEE Signal Process. Lett. 31, 1469–1473. doi: 10.1109/LSP.2024.3399668

[B49] Wilkes P. Lau A. Disney M. Calders K. Burt A. de Tanago J. G.. (2017). Data acquisition considerations for terrestrial laser scanning of forest plots. Remote Sens. Environ. 196, 140–153. doi: 10.1016/j.rse.2017.04.030

[B50] Xu X. Wang P. Gan X. Sun J. Li Y. Zhang L.. (2022). Automatic marker-free registration of single tree point-cloud data based on rotating projection. Artif. Intell. Agric. 6, 176–188. doi: 10.1016/j.aiia.2022.09.005

[B51] Yang B. Dong Z. Liang F. Liu Y. (2016). Automatic registration of large-scale urban scene point clouds based on semantic feature points. Isprs-J. Photogramm. Remote Sens. 113, 43–58. doi: 10.1016/j.isprsjprs.2015.12.005

[B52] Yang R. Li J. Liu Q. Huang W. Yin K. Qiao X.. (2020). Gradient-based method for the identification of multi-nodes in sugarcane. Inf. Process. Agric. 7, 491–499. doi: 10.1016/j.inpa.2020.01.004

[B53] Yang H. Shi J. Carlone L. (2020). Teaser: fast and certifiable point cloud registration. IEEE Trans. Robotics 37, 314–333. doi: 10.1109/TRO.2020.3033695

[B54] Yang T. Ye J. Zhou S. Xu A. Yin J. (2022). 3d reconstruction method for tree seedlings based on point cloud self-registration. Comput. Electron. Agric. 200, 107210. doi: 10.1016/j.compag.2022.107210

[B55] Zang Y. Meng F. Lindenbergh R. Truong-Hong L. Li B. (2021). Deep localization of static scans in mobile mapping point clouds. Remote Sens. 13, 219. doi: 10.3390/rs13020219

[B56] Zhang Q. Chen Z. Zhou Z. Wang L. Liao Q. Yang C.. (2024). 3d terrestrial lidar for obtaining phenotypic information of cigar tobacco plants. Comput. Electron. Agric. 226, 109424. doi: 10.1016/j.compag.2024.109424

[B57] Zhang C. Serra S. Quirós-Vargas J. Sangjan W. Musacchi S. Sankaran S. (2023). Non-invasive sensing techniques to phenotype multiple apple tree architectures. Inf. Process. Agric. 10, 136–147. doi: 10.1016/j.inpa.2021.02.001

[B58] Zhang W. Shao J. Jin S. Luo L. Ge J. Peng X.. (2021). Automated marker-free registration of multisource forest point clouds using a coarse-to-global adjustment strategy. Forests 12, 269. doi: 10.3390/f12030269

[B59] Zhou S. Kang F. Li W. Kan J. Zheng Y. (2020). Point cloud registration for agriculture and forestry crops based on calibration balls using kinect v2. Int. J. Agric. Biol. Eng. 13, 198–205. doi: 10.25165/j.ijabe.20201301.5077

[B60] Zhou G. Wang B. Zhou J. (2014). Automatic registration of tree point clouds from terrestrial lidar scanning for reconstructing the ground scene of vegetated surfaces. IEEE Geosci. Remote Sens. Lett. 11, 1654–1658. doi: 10.1109/LGRS.2014.2314179

[B61] Zhu L. Guan H. Lin C. Han R. (2022). Leveraging inlier correspondences proportion for point cloud registration. arXiv preprint arXiv:2201.12094.

